# Increasing the completion rate of the advance directives in primary care setting – a randomized controlled trial

**DOI:** 10.1186/s12875-021-01473-1

**Published:** 2021-06-18

**Authors:** Cunzhi Xu, Shi Yan, Jade Chee, Emily Pui-Yan Lee, Han Wei Lim, Sarah Woon Ching Lim, Lian Leng Low

**Affiliations:** 1 Singapore Health, Services, 31 Third Hospital Ave, #03-03 Bowyer Block C, Singapore, 168753 Singapore; 2 My Family Clinic (Punggol Central), 301 Punggol Central #01-02, Singapore, 820301 Singapore; 3 Department of Family Med & Continuing Care, Singapore General Hospital, 20 College Road, Singapore, 169856 Singapore

**Keywords:** Advance Directives, Primary Health Care, Randomized Controlled Trial

## Abstract

**Background:**

The completion rate of Advance Directives (ADs) has been low. This study aims to examine the effectiveness of two interventions 1) active counseling sessions coupled with passive patient education pamphlets, and 2) patient education pamphlets alone, compared with 3) control group (usual care), in increasing the completion rates of ADs in the primary care setting.

**Methods:**

Multicenter randomised controlled trial in four public primary care clinics in Singapore under Singapore Health Services. Randomization was performed via block randomization with Sequential Numbered Opaque Sealed Envelopes. Participants were randomized into 1) active intervention group (both counseling by primary care physicians and patient education pamphlets) or 2) passive intervention group (only patient education pamphlets), and 3) control group (usual care) with follow-up at 6 weeks. The main outcome measure is the proportion of participants who completed / planned to complete) ADs six weeks post-intervention.

**Results:**

Four hundred five participants were eligible to participate in the study. One hundred eighty-eight participants were recruited into the study (response rate = 46.4%), of which 158 completed the study. There was no significant difference between the control group, passive intervention group, and active intervention group, in terms of completion rates of ADs (29.4, 36.4, and 30.8% respectively).

**Conclusions:**

This randomized controlled trial did not support the use of patient education pamphlets with or without active counseling sessions in increasing the completion of ADs in a primary care setting in Singapore. The optimal intervention strategy depends on each health system’s context and resources, taking into consideration patients’ profiles, which deserves further studies.

**Trial registration:**

Registered on April 17, 2018 clinicaltrials.gov (NCT03499847).

**Supplementary Information:**

The online version contains supplementary material available at 10.1186/s12875-021-01473-1.

## Background

An Advance Directive (AD) is a legally binding instruction about a person’s future medical care in advance, in the event he or she later becomes unable to participate in decision making process about his or her care [1]. It promotes patients’ autonomy and patient empowerment based on each individual’s personal values and perceptions, cultural background, and goals and expectations of care [2, 3]. Since its conception, ADs have been widely promoted and supported worldwide as a critical part of Advance Care Planning (ACP). In the United States, Congress passed Patient Self Determination Act in 1991 that required healthcare facilities to inquire about ADs on a statutory basis [4].

However, the completion rate of ADs has been low [5–8], despite its well documented positive impact on patient care, including better satisfaction with physicians and clinic visits [9], decreased healthcare cost and utilization [10], decreased chances of demise in the hospital and use of life-sustaining treatment [11]. Population-based study estimated the rate of completed ADs ranged from 5—15% in the US [12]. This obvious contrast has led to many studies exploring potential barriers to completion of ADs, including low awareness and lack of knowledge of ADs from patients [13, 14] and lack of dedicated time by physicians [15]. These findings have led to efforts in developing effective solutions to promote completion of ADs. Several interventional studies have demonstrated success and shown that patient education and communication can be effective in promoting AD completion [16–19]. A recent systematic review on various interventions used to promote end-of-life planning suggested that the most effective method to increase the uptake of such plans is the combination of informative material and repeated conversations over clinical visits [20].

Primary care settings provide great opportunities for interventional efforts to address the issue of low AD completion rates where majority of patients receive their usual care. Primary care physicians’ training uniquely emphasizes holistic care, coordination of care, and excellent communication and thus they are best prepared to discuss ADs [3, 9]. It was also reported that primary care patients have high willingness to have ADs and both the young and the healthy subgroups expressed at least as much interest in planning ADs as those older than 65 and those in fair-to-poor health [21]. Patients also want their primary care doctors to initiate ACP earlier in the patient-physician relationship, earlier in their disease process, and while they are still in good health [22, 23]. Thus, it is imperative to develop effective strategies to increase the completion of ADs in primary care setting. A systematic review in 2007 on interventions to increase AD completion in the primary care setting showed that successful interventional programs often involved direct patient–health care professional interactions in iterative interactions over multiple visits whereas passive patient education materials may be ineffective [24].

An important gap in the current medical literature is that the existing intervention studies on completion of ADs in primary care settings were mostly in western countries. To the best of our knowledge, no randomised controlled trial has been conducted in Asia. We hypothesized that active counseling would similarly increase the completion rate of ADs in Singapore because AD is a comprehensive dialogue and process about a person’s health preferences in end-of-life scenarios, and direct interactions between individuals and health care professionals provide them with the opportunity to clarify their queries and offer assistance during the process of discussing and filling in an AD; however, it is well known that discussions on end-of-life issues are heavily influenced by cultural and societal factors, and there is no prior evidence on whether the above-mentioned strategies to improve completion of ADs may work in an Asian society with different ethnic and cultural backgrounds, and social values and philosophies. Singapore is a multi-ethnic, multicultural urban city state in Southeast Asia that faced the similar issue of low AD completion rate. Although Advance Medical Directive (AMD) Act was passed in Singapore Parliament in May 1996, its uptake by the public remains low [25].

In this randomized controlled trial conducted in Singapore, we aim to examine the effectiveness of two interventional strategies, namely 1) active counseling sessions coupled with passive patient education pamphlets, and 2) patient education pamphlets alone, compared with 3) control group (usual care), in increasing the completion rates of ADs in the primary care setting.

## Methods

### Study design

A three-arm randomized control trial was conducted for comparison between 1) active intervention group (both counseling by primary care physicians and patient education pamphlets), 2) passive intervention group (only patient education pamphlets), and 3) control group (usual care). The counseling by primary care physicians has two components. Firstly, primary care physicians counsel the patients in clinics. The counseling is structured according to the official public material (pamphlets) produced by the Ministry of Health, Singapore which covers concept of and terms surrounding ADs, the process of making ADs, and closely linked concepts such as terminal illness and palliative care [26] (Supplementary File 3). The pamphlets are in all four major languages used by Singapore residents (English, Mandarin, Malay and Tamil). Secondly, participants were given time for open-ended discussions and clarifications. Participants were given the pamphlets at the end of the counseling sessions. Participants in the passive intervention group were only given the pamphlets during their routine clinic consult by their primary care physicians but no active counseling on ADs were conducted.

Randomization was performed via block randomization with Sequential Numbered Opaque Sealed Envelopes (SNOSE), in variable blocks of 6 and 9, in a ratio of 1:1:1. At 6-weeks follow-up, phone recall was performed by a blinded member of the study team to assess the study outcomes (see *Outcome Measures*). A participant was considered a non-responder if the individual was unable to be contacted by phone call after 3 attempts on separate days or had requested to withdraw from the study prior to the 6-weeks mark. The study was conducted in accordance of the CONSORT 2010 checklist for randomized controlled trials (Suplementary File 1) [27].

Participants were recruited during routine clinic visits from four polyclinics in Singapore (Bedok, Marine Parade, Outram, Tampines). Polyclinics are large public primary care clinics located throughout Singapore that provide subsidized primary care [28]. Patients first attended their regular clinic visits during which primary care physicians invited the eligible patients to participate in the study and explained the trial as per trial protocol (Supplementary File 2). Interventions were conducted during the same clinic visit if patients gave consent to participate in the study. All participants were aged > 40 years and were patients of the aforementioned polyclinics. Exclusion criteria were known history of mental illness including depression and dementia, known diagnosis of terminal illness, and participants who had previously signed an AD or undergone ACP discussions. Trial duration is 29 March to 19 Dec 2018. Briefing sessions were conducted with the participating primary care physicians to ensure the adherence to the study design and protocol prior to recruiting patients. We also ensured consistency of the interventions in our study by the use of a checklist of discussion points based on the official public material (pamphlets) produced by the Ministry of Health, Singapore (Supplementary File 3).

Data on participants’ past medical history were obtained through electronic medical records. Baseline data of all participants were collected during the enrolment. Informed consent was obtained prior to enrolment.

### Sample size

Preliminary calculations suggested that 156 participants were required to have an 80% chance of being detected as significant at the 5% level, an increase in the primary outcome measure from 2.3% in the control group to 18.6% in the experimental group [29]. A sample size of 189 was planned for this study, taking into account a 20% drop-out rate.

### Outcome measures

The primary outcome of the study was the proportion of participants who completed or planned to complete the ADs at 6-weeks post-intervention (“completion of ADs”). A blinded member of the study performed a phone recall to each study participant to ask whether they have formally completed an AD with The Registry of Advance Medical Directives, Ministry of Health, Singapore or planned to complete an AD (e.g., in the process of filling in an AD but not formally processed by the Registry yet). If the participant reported a positive response, it would be counted as a positive outcome for that participant. The primary outcome was defined as a composite outcome of completed and planned AD because the Advance Medical Directive Act in Singapore mandates that an AD document is only valid when it is registered with the Registrar of Advance Medical Directives in The Registry of Advance Medical Directives, Ministry of Health Singapore. The Registrar will send the maker of the directive an acknowledgement when the directive has been registered [30]. Recognizing that the formal process may take variable amount of time and our overall objective is to assess whether our interventions had any influence in participants’ behaviors towards AD, we decided to include planned AD as a positive outcome.

Amongst the participants who reported a positive response, we explored the reasons behind their decision using the questions developed from previous publication [14]. The same was done for the other group with negative response to explore the common barriers to completion of the ADs [14].

### Statistical analysis

Fisher’s exact test was used to analyse primary outcomes, with p values of < 0.05 considered statistically significant. Statistical analysis was performed using Stata version 13.1. Exploratory sub-group analysis stratified by education level was planned prior to examine whether education level is a significant factor that influences likelihood of completing/planning to complete an AD. The reasons for and against ADs amongst participants were presented as descriptive data.

## Results

### Enrollment and follow-up

**Fig. 1:**
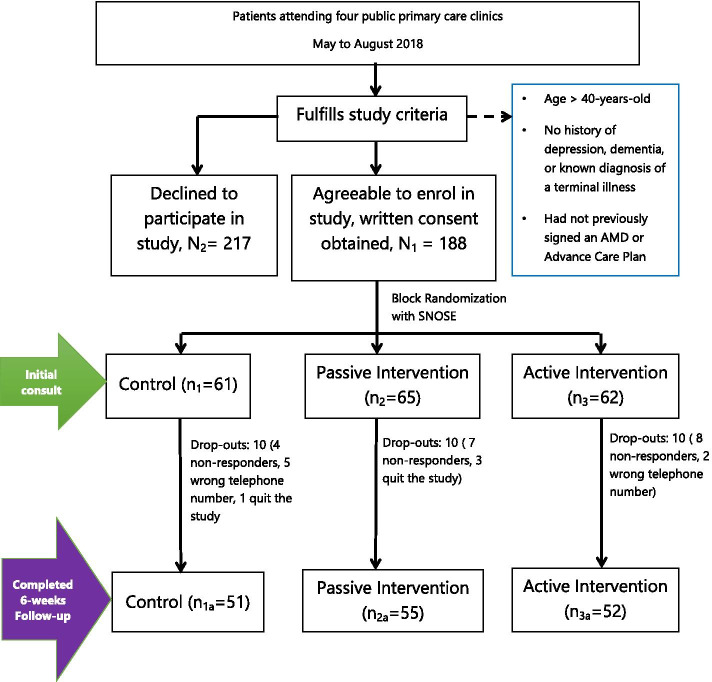
Study flowchart of participants

Out of 405 eligible participants, a total of 188 participants were recruited into the study (response rate = 46.4%), of which 158 completed the study. Of the 30 participants who did not complete the study, 4 dropped out from the study, while 26 were non-responders at the 6-week follow-up mark (Fig. 1).

### Baseline demographics

The baseline demographics of the study participants are presented in Table 1

**Table 1:** Baseline demographics of study participants

Demographic		Overall (n, %)	Control group (n, %)	Passive intervention group (n, %)	Active intervention group (n,%)
**Gender**	Males	84(53.2%)	25(49.0%)	33(60.0%)	26(50.0%)
	Females	74(46.8%)	26(51.0%)	22(40.0%)	26(50.0%)
**Religion**	Buddhism	54(34.2%)	17(33.3%)	20(36.4%)	17(32.7%)
	Taoism	15(9.5%)	3(5.9%)	7(12.7%)	5(9.6%)
	Christianity	36(22.8%)	13(25.5%)	10(18.2%)	13(25.0%)
	Islam	27(17.1%)	8(15.7%)	9(16.4%)	10(19.2%)
	Hinduism	4(2.5%)	1(2.0%)	2(3.6%)	1(1.9%)
	Others	6(3.8)	2(3.9%)	1(1.8%)	3(5.8%)
	No religion	16(10.1%)	7(13.7%)	6(10.9%)	3(5.8%)
**Marital status**	Single	18(11.4)	6(11.8%)	8(14.5%)	4(7.7%)
	Married	125(79.1)	42(82.4%)	40(72.7%)	43(82.7%)
	Widowed	7(4.4%)	1(2.0%)	4(7.3%)	2(3.8%)
	Divorced	8(5.1%)	2(3.9%)	3(5.5%)	3(5.8%)
**Education level**	Informal	10(6.3%)	4(7.8%)	5(9.1%)	1(19.9%)
	Primary	34(21.5%)	9(17.6%)	12(21.8%)	13(25.0%)
	Secondary	56(35.4%)	16(31.4%)	22(40.0%)	18(34.6%)
	Vocational	6(3.8%)	2(3.9%)	2(3.6%)	2(3.8%)
	High school	22(13.9%)	7(13.7%)	5(9.1%)	10(19.2%)
	University	24(15.2%)	11(21.6%)	8(14.5%)	5(9.6%)
	Not sure	6(3.8%)	2(3.9%)	1(1.8%)	3(5.8%)
**Employment status**	Full-time	61(38.6%)	19(37.3%)	19(34.5%)	23(44.2%)
	Part-time	16(10.1%)	6(11.8%)	7(12.7%)	3(%5.8)
	Retired	70(44.3%)	23(45.1%)	25(45.5%)	22(42.3%)
	Homemaker	11(7.0%)	3(5.9%)	4(7.3%)	4(7.7%)
**Number of chronic conditions**	0	21(13.3%)	9(17.6%)	7(12.7%)	5(9.6%)
	1 to 2	72(45.6%)	22(43.1%)	24(43.6%)	26(50.0%)
	3 or more	65(41.1%)	20(39.2%)	24(43.6%)	21(40.4%)

### Study outcomes

There was no significant difference in terms of completion rates of ADs between control group (29.4%), passive intervention group (36.4%), and active intervention group (30.8%), as shown in Table 2 (Fisher exact test *p* = 0.752).

**Table 2:** Completion rates of ADs in control and intervention groups

**Study group**	**Total**	**Completed / plan to complete ADs** (N, (%))	**Not completed and no plan to complete ADs** (N, (%))
Control group	51	15(29.4%)	36(70.6%)
Passive intervention group	55	20(36.4%)	35(63.6%)
Active intervention group	52	16(30.8%)	36(69.2%)
Overall	158	51(32.3%)	107(67.7%)

Fisher exact, *p* = 0.752

Exploratory sub-group analysis stratified by education level showed participants who had post-secondary school education (*n* = 52) compared with participants who had secondary school education or less (*n* = 100), had higher likelihood of completing/planning to complete an AD (42.3% vs 26.0%), (Fisher exact test *p* < 0.05).

Among participants who completed / planned to complete an AD (regardless of which intervention groups or control group they were randomized to), top reasons cited were 1) Wish to avoid prolonged suffering 2) Belief that passing away from a terminal illness is better than an artificially prolonged life 3) Acceptance of death in terminal illness (Supplementary Table 1).

Among participants who did not complete / plan to complete an AD (regardless of which intervention groups or control group they were randomized to), top reasons cited were 1) Unlikely to be in a situation that requires an AD 2) Inconvenience 3) Too young to be concerned about death (Supplementary Table 2).

## Discussion

### Summary

Our study suggests that passive patient education with or without active counseling session may not have a significant effect on completion rates of ADs in the primary care setting in Singapore. To the best of our knowledge, this is the first prospective randomized controlled trial in Asia to investigate the effectiveness of the counseling session and/or provision of patient education pamphlet in increasing the uptake of the ADs in the community setting.

### Strengths and limitations

A major strength of this study is that it is a multicenter, prospective randomized controlled trial which offers high level of evidence. Recruitment of participants from multiple primary care centers improved the representativeness of our samples. Randomization minimized the influence of factors other than intervention itself on study outcomes. The novelty of this study is from its study setting in a culturally different society in Asia compared to most other similar studies in Western countries. We also ensured consistency of the interventions in our study by the use of a checklist of discussion points based on the official Singapore AD booklet.

One of the limitations of this study is its potential selection bias. Out of the initial eligible pool of 405 patients, 188 patients agreed to be enrolled into this study. Each counseling session may vary in terms of duration and quality, which we attempted to mitigate by providing a checklist of tasks during each counseling session. The duration of the study may also be too short to detect the effects of the interventions. Another limitation is the possible contamination of the control (usual care) group. Notably, positive outcome in control group was higher than expected. In 2015, the number of registered AMDs in Singapore was 20,482 (the resident population was 4,949,465 in 2015) [31–34], whereas in our control group, positive outcomes were reported in 29.4% of participants. One of the possible reasons is that some primary care physicians may have seen participants in the active intervention group and subsequently participants in the control group where they may inadvertently “contaminated” the control group by providing active counseling subconsciously to control group patients during the routine clinic consult. We mitigated this issue by holding briefing sessions with the participating primary care physicians to ensure the adherence to the study design and protocol prior to recruiting patients. On the other hand, participants in the control group may have taken their own initiatives to complete / plan to complete an AD without primary care physicians’ counseling, hence leading to positive outcomes. In other words, the enrolment into the study itself was actually an unintended intervention to our participants in the control group, because they were briefed on the concepts and potential benefits of ADs during the recruitment and consent taking process.

### Comparison with existing literature

Existing literature on the effectiveness of the counseling and/or patient education pamphlets showed mixed results in increasing uptake of ADs in the primary care in the western countries. For example, Brown et all demonstrated mailing of patient education materials substantially increased completion of an AD [35] Sach et al. on the other hand, showed that counseling and an information booklet did not significantly increase a documented AD [36]. It was previously unknown whether similar intervention strategies to improve completion of ADs would be effective in an Asian setting because discussions on end-of-life issues are heavily influenced by cultural and societal factors. Our current study addressed this critical gap by providing strong evidence that passive patient education with or without active counseling session may not have a significant effect on completion rates of ADs in an Asian society. This may be explained by the heterogeneity in the exact scope and design of interventions used. For example, for patient education material, one study mailed a work book to patients that included case scenarios of ADs [19], whereas another study mailed videos to educate patients about ADs [35], yet several other studies chose to provide educational materials at the time of the visit [37, 38]. For active interventions, some were performed by physicians [36] whereas other studies engaged social workers [19, 39]. Counseling duration varied from 10 to 30 min [19, 39]. Some counseling sessions were done in single sessions while others spanned over multiple visits [40]. Furthermore, even with the same design of the intervention programs, there may be variability in the quality of interventions delivered.

Previously, many studies on ADs and ACP focused on patients of advanced age, under palliative care, or with terminal diseases [41–45]. Tools such as Serious Illness Conversation Guide were also developed and used by hospitalists to facilitate the process of ACP for patients with serious illness [46, 47]. However, ACP should be accessible to the general population in the community – anyone who wishes to engage in such conversations regardless of individuals’ health status [48]. Having ACP conversations allows an individual, even when he/she is young and healthy, to share personal values and beliefs and explore care preferences. Previous studies have demonstrated patients in primary care want to have ADs, regardless of age and health status, done with their primary care physicians [21–23]. Individuals with prior engagement in ACP were more likely to have better experiences with healthcare near the end of life, greater concordance between their care preferences and the actual care they receive, and lower stress amongst care givers [11, 16, 49]. Given that ACP is an ongoing conversations and process of engaging patients in their care arrangement, primary care physicians are uniquely advantaged to initiate ACP conversations because of their longitudinal therapeutic relationships with their patients and their comprehensive understanding of patients’ health priorities and social circumstances [50]. Some experts argued primary care physicians are in the best position to introduce and start the conversation regarding ACP [51, 52]. Our paper serves to stimulate further research on what is the most suitable form of intervention to promote ACP in an Asian primary care setting.

### Implications for research and/or practice

In our study, patient education pamphlets with or without active counseling sessions did not result in significantly higher completion rates of ADs in primary care settings in Singapore. This randomized controlled trial did not support the use of such strategy in increasing the completion of ADs in primary care setting in Singapore. A new set of strategies for such intervention programs aiming at increasing the completion of ADs in primary care setting in an Asian society may be needed. Traditional Chinese Singaporeans may believe in the superstition that talking about death is inauspicious and therefore intervention programs that involve discussion about end-of-life treatment and care preferences require careful design and need to be done with cultural sensitivity from physicians [53]. Another characteristic of Singapore local society is its traditional Confucian-influenced principles where the family unit, as opposed to individuals, plays a pivotal role in daily lives with filial piety emphasized and deeply rooted in the local culture [54]. Clinical settings and AD discussions are not exempt from this influences where an individual is often regarded as a part of a familial and social network with obligations towards the family, thereby promoting the influence of family caregivers in clinical decision-making [55–57]. Future studies in such cultural setting may consider Intervention programs that involve close family members which may be more effective in promoting completions of ADs in Asia. In an exploratory study conducted in Singapore, family unit was considered as the point of access to the patient and who knew the patient best, and that involving the family early in AD discussions and shared decision-making were frequently cited as key for successful uptake[58].

We also attempted to explore the reasons behind patients’ decisions to complete or decline the ADs. The top reasons among individuals who signed/planned to sign the ADs echoed the participant perceptions in Tay et al.’s study, whereby 87% of participants felt that passing away from terminal illness was better than an artificially-prolonged life; and significant persuading factors included avoiding prolonged suffering (89.6%) and acceptance of death (86.3%) [14]. The top three reasons for declining to sign ADs were also in agreement of the previous study [14]. This information may be used to further strengthen and improve the intervention content by incorporating more relevant points into the patient education materials and patient-physician discussions.

## Conclusion

This randomized controlled trial did not support the use of patient education pamphlets with or without active counseling session in increasing the completion of ADs in primary care setting in Singapore. The optimal intervention strategy depends on each health system’s context and resources, taking into consideration patients’ profiles, which deserves further studies.

## Supplementary Information

**Additional file 1.**

**Additional file 2.**

**Additional file 3.**

**Additional file 4.**

**Additional file 5.**

## Data Availability

The datasets used and/or analysed during the current study are available from the corresponding author on reasonable request.

## Abbreviations

AD: Advance Directives
AMD: Advance Medical Directive
