# Supplementary material for: Increasing the completion rate of the advance directives in primary care setting – a randomized controlled trial
Source: BMC Fam Pract. 2021 Jun 18;22:115. doi: 10.1186/s12875-021-01473-1 (PMC8214280; doi:10.1186/s12875-021-01473-1)
Supplement: Supplementary file 2 — Additional file 2. [file 12875_2021_1473_MOESM2_ESM.docx]

**
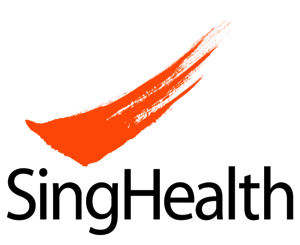
**

**STUDY PROTOCOL**

| **PROTOCOL TITLE:** |
| --- |
| Assessing the Effectiveness of Different Interventions in Increasing the Completion Rates of the Advanced Medical Directive in the Polyclinic Setting in Singapore |
|  |
| **PRINCIPAL INVESTIGATOR:** |
| Dr Low Lian Leng  Associate Consultant  Department of Family Medicine and Continuing Care  Singapore General Hospital |
| **CO-INVESTIGATORS:** |
| Dr Jade Nicolette Chee, Resident, Family Medicine Residency, Singhealth  Dr Cunzhi Xu, Resident, Family Medicine Residency, Singhealth |
|  |
| **STUDY TEAM MEMBERS:** |
| Dr Chui Yin Yap, Resident, Family Medicine Residency, Singhealth  Dr Wei Na Lai, Resident, Family Medicine Residency, Singhealth |
| Dr Emily Pui-Yan Lee, Resident, Family Medicine Residency, Singhealth  Dr Han Wei Lim, Resident, Family Medicine Residency, Singhealth  Dr Eunice Si Ying Cheong, Resident, Family Medicine Residency, Singhealth  Dr Yufei Li, Resident, Family Medicine Residency, Singhealth |
|  |
| Table of Contents  1. BACKGROUND AND RATIONALE 4  2. HYPOTHESIS AND OBJECTIVES 4  3. EXPECTED RISKS AND BENEFITS 4  4. STUDY POPULATION 5  4.1. List the number and nature of subjects to be enrolled. 5  4.2. Criteria for Recruitment and Recruitment Process 5  4.3. Inclusion Criteria 5  4.4. Exclusion Criteria 5  5. STUDY DESIGN AND PROCEDURES/METHODOLOGY 5  6. SAFETY MEASUREMENTS 6  6.1. Definitions 6  6.2. Collecting, Recording and Reporting of Adverse Events and Serious Adverse Events to CIRB 6  6.3. Safety Monitoring Plan 7  6.4. Complaint Handling 7  7. DATA ANALYSIS 7  7.1. Data Quality Assurance 7  7.2. Data Entry and Storage 7  8. SAMPLE SIZE AND STATISTICAL METHODS 7  8.1. Determination of Sample Size 7  8.2. Statistical and Analytical Plans 7  9. DIRECT ACCESS TO SOURCE DATA/DOCUMENTS 7  10. QUALITY CONTROL AND QUALITY ASSURANCE 8  11. ETHICAL CONSIDERATIONS 8  11.1. Informed Consent 8  Consent will be taken by the research co-ordinators in various communities during eye screening after explaining the study, obtaining voluntary agreement of the partcipant, explaining the number of visits and alternatives and after the participant is clear of his/her study rights. 8  11.2. Confidentiality of Data and Participant Records 8  12. PUBLICATIONS 8  13. RETENTION OF TRIAL DOCUMENTS 8  14. FUNDING and INSURANCE 8  15. References 9  16. Appendix 10 |
|  |
|  |
|  |

**PROTOCOL SIGNATURE PAGE**

Assessing the Effectiveness of Different Interventions in Increasing the Completion Rates of the Advanced Medical Directive in the Polyclinic Setting in Singapore

Sponsor Name: N/A

Declaration of Investigator

I confirm that I have read and approved the above-mentioned protocol and its attachments. I agree to conduct the described trial in compliance with all stipulations of the protocol, regulations and Singapore Guideline for Good Clinical Practice (SGGCP).

| Principal Investigator Name:  _________Dr Low Lian Leng_________________    Principal Investigator Signature:  _______________________________________    Date:  ________15/10/2017___________ |
| --- |

| **BACKGROUND AND RATIONALE** |  |
| --- | --- |
| An Advanced Medical Directive (AMD) is a legal document in Singapore that allows one to inform their medical practitioner that they do not want any life-sustaining treatment should they become unconscious and terminally ill where death is imminent. It may be completed by any person, of sound mind, aged 21 years and above. The AMD Act was passed in the Singapore Parliament in May 1996.  A cross-sectional, community-based study surveyed 1200 selected households in Singapore. Only **37.9%** of the sample population had heard of the AMD prior to the study, though when approached, **60.6%** were willing to sign an AMD^1^. As of 2017, less than 25,000 people (0.45%) in Singapore have opted to sign an AMD^2^.  Past studies have shown that end of life discussions reduce stress, anxiety, and depression in surviving relatives^3^. A local study in 2010 revealed a 33% patient-surrogate discrepancy in end-of-life decision making^4^.  There is a lack of intervention studies regarding AMD in Singapore. In recent years, the AMD has been the subject of much public discussion and debate, in light of Singapore’s ageing population, end-of-life planning and other related issues^1^.  In this study, we aim to compare the effectiveness of counselling sessions together with pamphlets, versus pamphlets alone, versus current care, in increasing the completion rates of the AMD in the Singapore Polyclinic setting. |  |
| **HYPOTHESIS AND OBJECTIVES** |  |
| We hypothesize that:   - Counselling with pamphlets will be more effective than pamphlets alone in increasing the uptake of the AMD. - Either intervention will increase the uptake of the AMD as compared to current care.   Primary outcomes:   - Number of patients who completed the AMD - Number of patient who expressed interest to complete the AMD   Secondary outcomes:   - Barriers to completion of AMD | |
| **EXPECTED RISKS AND BENEFITS** |  |
| This is a questionnaire-based study, no major risks are identified.  A lack of awareness is a frequently report reason for not completing an advance directive. In a study done by Tay et al in 2010, awareness of AMD in Singapore was 37.9%, though 60.6% among participants were willing to sign an AMD. Moreover, it was noted that high knowledge scores of the AMD of participants were associated with a greater willingness to complete an AMD.  While several intervention studies have shown that education and communication can be effective in promoting end-of-life discussion and planning, no such studies have been done in the primary care setting in Singapore.  We hope that through this study, we will be able to determine the most effective method in increasing the completion rates of the AMD in Singapore, thereby reducing stress, anxiety, and depression in surviving relatives. |  |
| **STUDY POPULATION** |  |
| List the number and nature of subjects to be enrolled. |  |
| 195 patients attending the Residency Continuity Clinic from January 2017 to May 2018 who fit the eligibility criteria will be enrolled and randomised into 3 arms: the active intervention group, the passive intervention group, and the control group. |  |
| Criteria for Recruitment and Recruitment Process |  |
| Informed consent will be obtained before initiation of the study. Potential participants will be approached during the Residency Continuity Clinic. Consent will be taken during clinic consultation in the consultation rooms. |  |
| Inclusion Criteria |  |
| - Age > 40 - Singhealth Polyclinics (Bedok, Marine Parade, Outram, Tampines) |  |
| Exclusion Criteria |  |
| - History of dementia - History of depression - Known diagnosis of terminal illness - Previously signed AMD or Advanced Care Planning |  |
| **STUDY DESIGN AND PROCEDURES/METHODOLOGY** |  |
| **Patient Enrolment:**  This is a randomised control trial consisting of 3 arms:-   1. **Active Intervention group (n=65)**    - Counselling provided    - Pamphlet given 2. **Passive Intervention group (n=65)**    - Pamphlet given 3. **Control group (n=65)**    - Neither counselling nor pamphlet given     **Randomization:**  Randomisation will be performed via block randomisation (total N=195) via Sequential Numbered Opaque Sealed Envelopes (SNOSE), in variable blocks of 6 and 9 (ratio of groups 1:1:1).    **Follow-up Assessment**  Post-intervention surveys will be conducted via telephone at 6 weeks by a blinded assessor.    A flowchart of the protocol is given in **Appendix 1**. |  |
| **SAFETY MEASUREMENTS** |  |
| Definitions |  |
| An adverse event (AE) is any untoward medical occurrence in a participant or clinical investigation subject administered a pharmaceutical product and which does not necessarily have a causal relationship with this treatment.  A serious adverse event (SAE) or reaction is any untoward medical occurrence that at any dose:   - Results in death - Is life-threatening - Requires inpatient hospitalisation or prolongation of existing hospitalisation - Results in persistent or significant disability/incapacity or - Is a congenital anomaly/birth defect - Is a medical event that may jeopardize the participant and may require medical or surgical intervention to prevent one of the outcomes listed above.   As this is a questionnaire-based study, no AEs or SAEs are expected. |  |
| Collecting, Recording and Reporting of Adverse Events and Serious Adverse Events to CIRB |  |
| Reporting of adverse events involves the PI submitting to the approving CIRB the completed SAE Reporting Form within the stipulated timeframe. PI is responsible for informing the institution representative (local SAE resulting in death), sponsor or regulatory bodies as required and appropriate.  Reporting timeline to CIRB:   - SAE that result in death, regardless of causality, should be reported immediately - within 24 hours of the PI becoming aware of the event. - Local life-threatening (unexpected/ expected) SAE should be reported no later than 7 calendar days after the Investigator is aware of the event, followed by a complete report within 8 additional calendar days. - Local unexpected SAE that are related events, but not life-threatening, should be reported no later than 15 calendar days after the investigator is aware of the event. - An increase in the rate of occurrence of local expected SAE, which is judged to be clinically important, should be reported within 15 calendar days after the PI is aware of the event. - Local expected SAE should be reported annually (together with Study Status Report for annual review). - Local unexpected and unlikely related SAE that are not life-threatening should also be reported annually (together with Study Status Report for annual review). - Local unexpected AE that are related events should be reported at least annually (together with Study Status Report for annual review). - Non-local unexpected SAE that are fatal or life threatening and definitely/probably/possibly related should be reported not later than 30 calendar days after the PI is aware of the event. |  |
| Safety Monitoring Plan N/A |  |
| Complaint Handling |  |
| Complaints will be handled by the research team and if required the Quality Assurance team at Singhealth. |  |
| **DATA ANALYSIS** |  |
| Data Quality Assurance |  |
| The Investigator(s)/ Singhealth will permit study-related monitoring audits, MCRC and or EC review and regulatory inspection(s), providing direct access to source data/ document. |  |
| Data Entry and Storage |  |
| Data will be stored in a private database in a stand-alone PC in each recruitment site's office under lock and key access. The principal investigator and co-investigators will have access to the data. This access can be monitored through the computer records. The data also will be coded and the participants will be de-identified and the master list will be locked with the password. De-identified data will also be stored in shared folders that can be accessed only by the research team members. |  |
| **SAMPLE SIZE AND STATISTICAL METHODS** |  |
| Determination of Sample Size |  |
| 156 patients are required to have an 80% chance of detecting as significant at the 5% level, an increase in the primary outcome measure from 2.3% in the control group to 18.6% in the experimental group^7^. The sample size calculation with adjustment made for 20% drop out rate is calculated to be 195^8^. |  |
| Statistical and Analytical Plans |  |
| - To assess primary outcomes of the number of patients who completed the AMD and the number who expressed interest to complete the AMD, Chi Square analysis or Fisher exact (categorical variables) will be used. - To assess secondary outcomes of barriers to completion of the AMD, descriptive anlysis will be used. |  |
| **DIRECT ACCESS TO SOURCE DATA/DOCUMENTS** The investigator(s)/institution(s) will permit study-related monitoring, audits and/or IRB review and regulatory inspection(s), providing direct access to source data/document. **QUALITY CONTROL AND QUALITY ASSURANCE** N/A **ETHICAL CONSIDERATIONS** This study will be conducted in accordance with the ethical principles that have their origin in the Declaration of Helsinki and that are consistent with the Singapore Good Clinical Practice and the applicable regulatory requirements.  This final study protocol, including the final version of the Participant Information and Informed Consent Form, must be approved in writing by the Centralised Institutional Review Board (CIRB), prior to enrolment of any participant into the study.  The principle investigator is responsible for informing the CIRB of any amendments to the protocol or other study-related documents, as per local requirement. |  |
| Informed ConsentConsent will be taken by the research team members after explaining the study protocol, obtaining voluntary agreement of the participant, explaining the number of visits and alternatives and after the participant is clear of his/her study rights. |  |
|  |  |
| Confidentiality of Data and Participant Records |  |
| Research data and participant database will be stored each recruitment site's office under lock and key access. Hard copies will be stored in Singhealth research office at Connection One, in a locked cabinet. |  |
|  |  |
| **PUBLICATIONS** |  |
| Please refer to the Singhealth publication policy. |  |
| **RETENTION OF TRIAL DOCUMENTS** |  |
| The research data will be stored for at least 15 years and then destroyed/deleted. |  |

| **FUNDING and INSURANCE** |
| --- |
| No funding was received for this study. |

**References**

1. Tay M, Chia SE, Judy Sng J. Knowledge, Attitudes and Practices of the Advance Medical Directive in a Residential Estate in Singapore. Ann Acad Med Singapore 2010;39:424-8.
2. Registry of Advance Medical Directives, Ministry of Health
3. Azaloulay E, Pochard F, Kentish-Barnes N, Chevret S, Aboab J, Adrie C, et al. Risk of post-traumatic stress symptoms in family members of intensive care unit patients. Am J Respir Crit Care Med 2005;171:987-94.
4. Foo AS, Lee TW, Soh CR. Discrepancies in end-of-life decisions between elderly patients and their named surrogates. Ann Acad Med Singapore. 2012 Apr;41(4):141-53.
5. Houben CH, Spruit MA, Groenen MT et al. Efficacy of advance care planning: a systematic review and meta-analysis. J Am Med Dir Assoc. 2014 Jul;15(7):477-89
6. Ramsaroop SD^1^, Reid MC, Adelman RD. Completing an advance directive in the primary care setting: what do we need for success? J Am Geriatr Soc. 2007 Feb;55(2):277-83.
7. Grimaldo DA, Wiener-Kronish JP, Jurson T et al. A randomized, controlled trial of advanced care planning discussions during preoperative evaluations. Anesthesiology. 2001 Jul;95(1):43-50.
8. Sealed Envelope Ltd 2012. Power calculator for binary outcome superiority trial. Available from: <https://sealedenvelope.com/power/binary-superiority/> [Accessed Thu Sep 21 2017].

**Appendix 1**
