# Supplementary material for: Increasing the completion rate of the advance directives in primary care setting – a randomized controlled trial
Source: BMC Fam Pract. 2021 Jun 18;22:115. doi: 10.1186/s12875-021-01473-1 (PMC8214280; doi:10.1186/s12875-021-01473-1)
Supplement: Supplementary file 3 — Additional file 3. [file 12875_2021_1473_MOESM3_ESM.pdf]

# Advance Medical Directive
# 预先医疗指示
# Arahan Awal Perubatan
# மருத்துவ முன் உத்தரவு

## What You Should Know
## 您所要了解的
## Apa Yang Anda Perlu Tahu
## உங்களுக்குத் தெரிய வேண்டியது

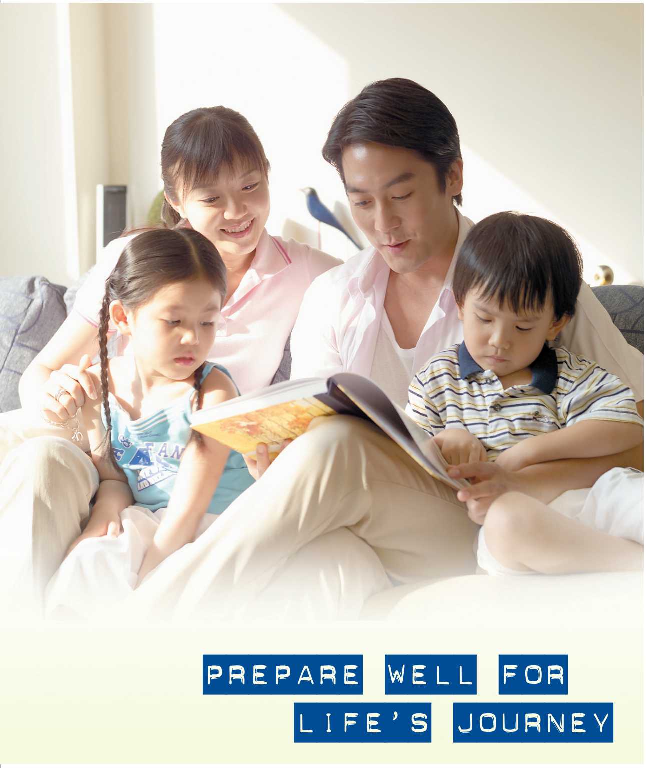


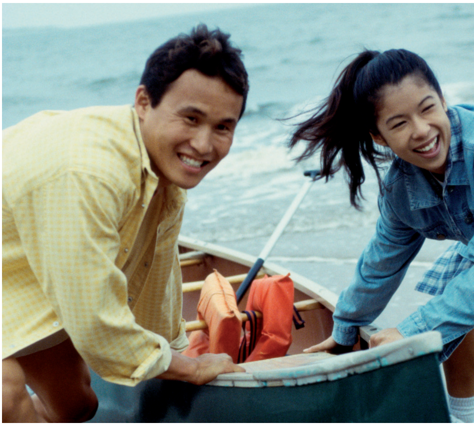

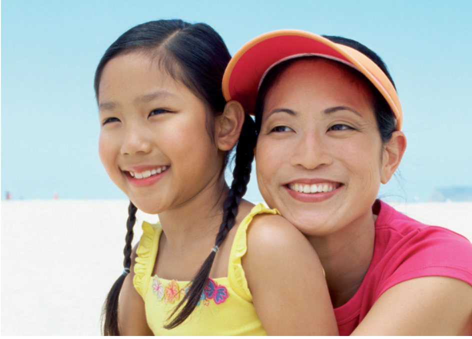

MINISTRY OF HEALTH
SINGAPORE

# Advance Medical Directive

# 预先医疗指示

# Arahan Awal Perubatan

# மருத்துவ முன் உத்தரவு

# *ADVANCE MEDICAL DIRECTIVE*

***New advances in medical knowledge and technology create new choices for both patients and health care providers. Some of these choices raise new ethical and legal issues.***

***One issue is that modern medical technology can technically prolong life in the final stages of a terminal illness. However, it cannot stop the dying process. In such situations, further medical intervention would be medically ineffective, and a decision has to be made whether to withdraw such futile medical intervention. Some terminally ill persons who are unable to express their wishes at that time, may want to be spared further suffering and be allowed to die naturally, in peace and with dignity.***

***The law in Singapore allows Singaporeans who wish to make an advance medical directive to do so. The Advance Medical Directive Act was passed in Parliament in May 1996. Before the AMD Act, people who had views on the medical treatment they wish to received when terminally ill, did not have the chance to make their wishes known. Thus, the decision of medical treatment was left to their doctor and family members.***

## TERMS AND DEFINITIONS

### 1. What is an Advance Medical Directive?

An Advance Medical Directive (AMD) is a legal document that you sign in advance to inform the doctor treating you (in the event you become terminally ill and unconscious) that you **do not want** any extraordinary life-sustaining treatment to be used to prolong your life.

Making an AMD is a **voluntary** decision. It is entirely up to you whether you wish to make one. In fact, it is a criminal offence for any person to force you to make one against your will.

### 2. What is meant by "terminal illness"?

**"Terminal illness"** is defined in the Act as an incurable condition caused by injury or disease from which there is no reasonable prospect of a temporary or permanent recovery. For such a condition, death is imminent even if extraordinary life-sustaining measures were used. These measures would only serve to postpone the moment of death for the patient.

### 3. What is meant by "extraordinary life-sustaining treatment"?

**"Extraordinary life-sustaining treatment"** is any medical treatment which serves only to prolong the process of dying for terminally ill patients but does not cure the illness.

An example is the respirator that is connected to a patient to assist him/her to breathe. It serves only to artificially prolong the life of a terminally ill patient.

## MAKING AN AMD

### 4. Who can make an AMD? How do I make an AMD?

Anyone who is 21 years old and above, and of sound mind can make an AMD. All you need to do is complete an AMD form, signed in the presence of two witnesses, and return it to the Registrar of AMDs.

### 5. Where can I get the form for making an AMD?

A form (Form 1) is included at the back of this booklet, which you can get at medical clinics, polyclinics and hospitals. You may also ask your doctor for the form if you have decided to make an AMD. Alternatively, you can also download the AMD form online at *http://www.moh.gov.sg/amdform*

N.B.: When printing out the form, please print it on both sides of one sheet of paper.

### 6. Do I need a lawyer to make an AMD?

No, you do not need a lawyer to make an AMD.

## WITNESSING OF AMD

### 7. Do I need to consult a doctor when making an AMD?

Yes. The doctor whom you consult will act as the first witness, whose responsibility is to ensure that:

(a) You are not being forced into making the AMD
(b) You are of sound mind
(c) You understand the nature and implications of making an AMD

### 8. Who can be a witness?

One of the two witnesses must be a doctor, preferably the patient's family doctor. The other witness must be at least 21 years old.

Both witnesses must not have any vested interests in the patient's death. If the witnesses are relatives, so long as they have no vested interests in the patients' death, they would be allowed to act as witness.

A doctor who for any reason objects to the AMD and registers his objection with the Registrar can refuse to witness the signing of an AMD. You can then approach another doctor to make your AMD.

### 9. Must both witnesses be present during the making of an AMD?

Yes, both witnesses must be present together to witness the person signing the AMD, as well as to sign the AMD form. As the first witness is your doctor, the second witness may be his nurse or any other suitable person.

### 10. Can I complete Form 1 before I see a doctor?

You can fill in your details on Form 1 first. However, the two witnesses and you must sign the form at the same sitting.

### 11. How much does it cost to make an AMD?

To make an AMD, you will have to consult a doctor who, as a witness, is required to explain the AMD to you. You may have to pay the doctor for the services rendered. However, the AMD form itself is free.

### 12. Can I make an AMD if I do not have a terminal illness? Should I inform my family?

It is best to make an AMD when there is no pressure to do so - when you are well and healthy. If your family opposes it, you may wish to obtain their understanding before making the AMD. If you do not wish to inform your family, you can still proceed to make an AMD, which will remain confidential, until you wish to disclose it to someone.

### 13. Is it compulsory for every adult in Singapore to make an AMD?

No, it is **NOT** compulsory. The AMD is valid only when it is made voluntarily. No one can force another person to make an AMD.

### 14. Can a parent make an AMD on behalf of his/her child? Can a parent or sibling make an AMD on behalf of a mentally incapable person?

No one can make an AMD on behalf of another person, whether child or adult.

### 15. Can my family members overrule or object to my AMD being carried out?

If you have made an AMD, regardless of objections from members of your family, the doctors will have to respect your AMD. Hence, while making an AMD is a very personal decision, we encourage you discuss it with your family members and loved ones. This will help them understand and respect your wishes should you become terminally ill.

### 16. How can we be sure that nobody, including the illiterate, is misled into signing an AMD?

It is the doctor's responsibility to fully explain and discuss the matter with the person and to ensure that he/she fully understands what he/she is signing.

**Forcing or misleading another person into signing an AMD is an offence punishable under the law.** If convicted, such a person can be fined and imprisoned and disqualified as a beneficiary.

### 17. Will the hospital know if I have made an AMD?

Hospital staff, including doctors and nurses do not know who has made an AMD as it is confidential, and they are **NOT** allowed to ask you if you have made an AMD. Hence, you are encouraged, to inform the hospital staff if you have made an AMD. However, if your attending doctor has reasons to believe that you are terminally ill and unable to make your wishes known to him, he can check with the Registrar of Advance Medical Directives on whether you have made an AMD.

### 18. Can I make an AMD while overseas?

As long as the first witness is a doctor currently registered with the Singapore Medical Council (SMC), and the AMD is filed with the AMD Registry in Singapore, the AMD is considered valid.

### 19. Can the AMD be carried out when I am overseas?

When you are overseas, the legal status of the AMD will depend on the local laws of the country where you are. Thus the AMD is usually only applied/effected when you are in Singapore.

## AMD VS EUTHANASIA

### 20. Is the AMD a type of euthanasia or mercy killing?

No, AMD is **NOT** euthanasia or mercy killing. Euthanasia/mercy killing is the deliberate ending of the life of a person suffering from an incurable and painful disease by unnatural means, such as the administration of lethal chemicals.

An AMD acts as advanced instruction for your doctor, not to prolong your life with extraordinary life-sustaining treatment, and to let the dying process take its natural course when you become terminally ill and unconscious.

### 21. Does the AMD Act encourage euthanasia?

No, the AMD Act does **NOT** encourage euthanasia. On the contrary, the AMD Act is explicitly and categorically against euthanasia. The Act states:

> ***Section 17(1) - Nothing in the Act shall authorise an act that causes or accelerates death as distinct from an act that permits the dying process to take its natural course.***
>
> ***Section 17(2) - It is hereby declared that nothing in this Act shall condone, authorise or approve abetment of suicide, mercy killing or euthanasia.***

A terminally ill patient **CANNOT** use the AMD to commit suicide, nor does the AMD allow doctors to assist in any suicide attempt. This is punishable by law.

## CERTIFICATION OF TERMINAL ILLNESS

### 22. Who can certify that a person is terminally ill?

Three doctors, including the patient's hospital doctor must unanimously certify a patient's terminal illness. Two of the doctors must be specialists.

### 23. What happens if the panel of doctors cannot reach an unanimous decision?

If the first panel of three doctors cannot agree unanimously that the patient is terminally ill, the doctor-in-charge will review his diagnosis. If he is still of the opinion that the patient is terminally ill, the matter will be referred to a second panel of three specialists, to be appointed by the Ministry of Health.

### 24. Can an AMD take effect if the second panel of doctors also cannot agree on the diagnosis?

No, the AMD **cannot** take effect if the second panel cannot agree unanimously that the patient is terminally ill. The patient's life will continue to be sustained and he will receive medical treatment as normal.

### 25. Do I have to pay for the services of the second panel of three specialists appointed by MOH?

You do not have to pay for their services.

## WHAT HAPPENS AFTER AN AMD IS MADE?

### 26. Must I inform my doctors or anyone that I have made an AMD?

You are not required to disclose your AMD to anyone. However, it is advisable that you discuss with your family and loved ones before making an AMD.

### 27. Who will know whether I have made an AMD?

The register of AMD will be kept confidential and will only be disclosed to:

i. individuals authorised by you in writing;
ii. your doctor, when he has reason to believe that you are terminally ill and incompetent in making your wishes known to him.

### 28. If a patient who has made an AMD suffers from terminal illness, will he/she be left without medical treatment or left to starve?

Doctors will continue to provide palliative care (i.e., treat and relieve pain and suffering of a patient with terminal illness).

Even if the AMD has taken effect, palliative care and medication will continue to be provided.

### 29. What is palliative care?

Palliative care helps to improve the quality of life of patients with terminal illness and their family members through prevention, assessment, and treatment of pain and other physical, psychological and spiritual problems.

The AMD Act specifies that palliative care includes:

- provision of reasonable medical procedures for the relief of pain, suffering or discomfort; and
- reasonable provision of food and water.

### 30. What safeguards are there to ensure that I am given all necessary treatment before the AMD is carried out?

It is the professional responsibility of every doctor to ensure that all necessary treatment be given as long as the patient is not certified terminally ill and there is a possibility of recovery. Even if the patient is considered terminally ill, the doctor has the responsibility to provide treatment to minimise pain and suffering.

This is the current practice, and will continue whether or not the patient has made an AMD.

# REVOKING AN AMD

## 31. If I change my mind about an AMD which I have made earlier, can I revoke it?

An AMD can be revoked at any time in the presence of at least one witness. The person making the revocation should do so by filling in **Form 3**, which is the standard form for revocation of an AMD. *(Those who have made an AMD will receive **Form 3** together with **Form 2**, which confirms that their AMD has been registered by the Registry of AMDs)*

Alternatively, the person or his witness could write a simple letter to the Registrar of AMD.

### The letter should contain the following information

(a) the name and NRIC of both the person revoking the AMD and the witness, along with their addresses and home and office telephone numbers

(b) time, date and place where the revocation was made

(c) if the letter is written by the witness, the method of communication which the person used to communicate his intention to revoke the AMD (e.g. orally, sign language). The witness should also state the reason why the person revoking the AMD could not do so himself.

Revocations should be sent to the Registry of AMDs as soon as possible.

## 32. What if my condition is such that I am unable to write?

In this situation, an AMD can be revoked orally or in any other way in which you can communicate. It is then the responsibility of the witness to the revocation to submit the notice of revocation using Form 3 or the letter as described above. **The witness should also state the reason why the person revoking the AMD could not do so himself.**

***Section 7(4) - It is the duty of the person "who has witnessed a revocation, whether made in writing, orally, or in any other way in which the patient can communicate, to inform the Registrar" of the revocation.***

## WHAT IF I DO NOT HAVE AN AMD?

It is not compulsory to make an AMD if you do not wish to do so. If a terminally ill patient who has not made an AMD, is unconscious and incapable of expressing his wishes, the current practice will continue. The doctor will consult the patient's family members to decide on the patient's treatment.

**Remember,** the making of an AMD is **not** a pre-requisite to gaining admission to any hospital. By law, it will not affect any insurance policy.

## NOTES FOR AMD FORM

1) The form for making the AMD (Form 1) is at the back of this booklet. You can also get it from your doctor, who must be your first witness. The form can also be downloaded from the MOH website: *http://moh.gov.sg/amdform*

2) Your doctor will explain the safeguards, procedures and when the AMD applies.

3) He will ensure that you are over 21 years, you are signing voluntarily, you understand the consequences of signing and are of sound mind.

   Indications of a sound mind are that you understand what you are signing and the implications, you can state your own name and that of your family members and you know the current time and place.

4) When the AMD form has been signed by you and the two witnesses, you can send it to the Registry of AMD, using the pre-paid envelope attached at the back of this booklet.

5) Once the AMD Registry has received your form, it will send you an acknowledgement letter **(Form 2)** to confirm that you have been registered in the AMD Registry.

For more information on Advance Medical Directives,
call the Ministry of Health AMD hotline at 63259136 or
visit the MOH website at

*http://www.moh.gov.sg/amd*

# *预 先 医 疗 指 示*

*医学知识及医疗科技的新进展，为病人和医务人员提供了新的选择，但也由此引发了新的道德和法律问题。*

*其中之一是，现代医疗科技能在技术上延长患绝症晚期病人的生命。然而这并无法阻止病人的死亡过程。在这种情况下，进一步的治疗在医学上是徒劳无功的，是否要停止这类徒劳无功的医疗，就必须作个决定。一些已无法表达自己意愿的绝症患者，也许不愿再忍受痛苦，他们希望可以自然地，安详地带着尊严离开这个世界。*

*那些希望立下预先医疗指示的国民，我国法律允许他们这么做。国会于1996年5月通过了预先医疗指示法令。法令实施前，人们没有机会表明自己一旦患上末期病症时所愿接受的医疗。于是，决定医疗的方法便落在他们的医生和家人身上。*

## 术语与释义

### 1. 预先医疗指示是什么？

预先医疗指示是一份由您事先签署的法律文件，它告诉您的主治医生（当您一旦患上不治之症且陷入昏迷状态时），您**不希望使用**任何维持生命的特别治疗以延长您的生命。

订立预先医疗指示是一项**自愿**的决定。如果这项决定是别人强迫您做的，这等于犯了刑事罪。

### 2. “绝症”是指什么？

在有关法令中，**“绝症”**是指由于受伤或疾病引起的不可能医治的状况，病人已失去暂时或永久康复的合理可能性。在这种情况下，即使是使用维持生命的特别方法，死亡也即将来临。这些维持生命的特别方法只能拖延死亡的时间。

### 3. “维持生命的特别治疗”是指什么？

**“维持生命的特别治疗”**是指只能延长患绝症病人的死亡过程，却不能治愈有关疾病的任何医疗方法。

人工呼吸器便是一个例子，它接在病人身上，帮助病人呼吸。其作用只是通过人工的方法延长垂危病人的生命。

## 预先医疗指示的订立

### 4.　谁能订立预先医疗指示？如何订立预先医疗指示？

凡年龄在21岁以上，且精神正常者，均能订立预先医疗指示，您只需填写一份预先医疗指示表格，在两名证人面前签名，然后把表格交给预先医疗指示注册官。

### 5.　我能够在哪里索取表格呢？

这本手册的背面附有一张表格（表格1）。您能够在私人诊所，综合诊疗所和医院索取这些手册。若您决定订立预先医疗指示，您也能够向医生索取表格。又或者，您可上网到*http://www.moh.gov.sg/amdform*下载表格。

注意：打印表格时，请印在一张纸的两面。

### 6.　我需要一名律师才能订立预先医疗指示吗？

不，您不需要一名律师才能订立一份预先医疗指示。

## 预先医疗指示的见证

### 7.　我需要咨询医生才能订立预先医疗指示吗？

需要。您所咨询的这名医生将会是您的首要证人，他的责任是确保：

(a) 您不是被他人强制订立预先医疗指示
(b) 您是在神智清醒的情况下作出决定
(c) 您清楚了解订立预先医疗指示的性质以及订立指示的含义

### 8.　谁能当证人？

两名证人中必须有一名是医生，最好是病人的家庭医生。另一名证人的年龄不得小于21岁。

两名证人都不能从病人的死受益。如果他们是亲戚，只要不会从病人的死受益，便能担任证人。

如果医生因任何理由反对担任预先医疗指示的证人并将他反对的理由向注册官备案，他可以拒绝担任证人。您可以另找一名医生协助您订立您的预先医疗指示。

## 9. 订立预先医疗指示时，两名证人是否必须同时在场？

是的，两名证人都必须一起在场见证预先医疗指示的签署，并在预先医疗指示表格上签名。由于第一证人是您的医生，第二位证人可由他的护士或任何其他合适的人选担任。

## 10. 我能够在咨询医生前填写表格1吗？

您可以事先在表格1填写您的个人资料。不过，您必须在两位证人的见证下才能签字。

## 11. 订立预先医疗指示要花多少钱？

您咨询的这名医生也兼作您的证人，所以他必须为您解释订立预先医疗指示的有关内容。您可能需要支付医生的服务费。但是，表格是免费的。

## 12. 我没患上绝症是否也可以订立预先医疗指示？我是否应该通知我的家人？

预先医疗指示最好是在没有压力的情况下，即当您身体健康的时候就订立。 如果家人反对，您可以在订立预先医疗指示之前，先取得他们的谅解。如果您不想通知您的家人，您仍然可以订立预先医疗指示，它将受到保密，直到您想向任何人透露为止。

## 13. 是否我国每个成人都必须订立预先医疗指示？

不，订立预先医疗指示**不是**强制性的。一份有效的预先医疗指示必须是在自愿的情况下设立的。任何人都不能强迫他人订立预先医疗指示。

## 14. 家长能否代孩子订立预先医疗指示？家长或兄弟姐妹能否代智障者订立预先医疗指示？

任何人都不能代他人订立预先医疗指示，无论是儿童还是成人。

## 15. 我的家人是否能拒绝我的预先医疗指示或对它的执行提出反对？

如果您不顾家人反对而立下了预先医疗指示，医生就必须尊重您的指示。有鉴于此，虽然订立预先医疗指示是个人的抉择，但我们还是鼓励您和亲人商量。这将有助于他们了解您的愿望，并万一在您生命垂危时，尊重您的意愿。

## 16. 我们如何确保没有人，包括不识字的人在内，会被误导立下预先医疗指示？

医生有责任向有关人士解释清楚，并与他进行全面的讨论，确保他完全了解他所签的是一份怎样的文件。

**强迫或误导他人签立预先医疗指示是犯法的，违法者将受到法律惩罚。**一旦罪名成立，他可能被判罚款和监禁，并被取消受益人资格。

## 17. 如果我订立了预先医疗指示，医院是否会知道？

医院职员，包括医生和护士，并不知道哪些人已经订立了预先医疗指示，因为这是保密的。他们也**不可以**问您是否已订立了预先医疗指示。因此若您已订立了预先医疗指示，我们鼓励您通知他们一声。但如果您的主治医生有理由相信您已患上末期绝症并无法向他表达您的意愿，他可以向预先医疗指示注册官查询您有否订立预先医疗指示。

## 18. 我可以在国外订立预先医疗指示吗？

只要首要证人是一名在新加坡医学评议会注册的合法医生，而且预先医疗指示是交给新加坡预先医疗指示注册局，这份预先医疗指示就算有效。

## 19. 我是否能在国外为我的预先医疗指示生效？

当您身在国外，它国的法律将决定您的预先医疗指示的合法资格。因此，预先医疗指示将会在您回国时才会生效。

# 预先医疗指示与安乐死

## 20. 预先医疗指示是否是一种安乐死或无痛苦致死术？

不是，预先医疗指示并**不是**安乐死或无痛苦致死术。安乐死或无痛苦致死术是用非自然方法，如施用致死药物，蓄意地结束正受折磨的绝症患者的生命。

预先医疗指示犹如预先给您的医生指示，一旦您患上绝症并陷入昏迷状态时，不要用维持生命的特别治疗来延长您的生命，而是让死亡的过程自然发生。

## 21. 预先医疗指示法令是否鼓励安乐死?

不是，预先医疗指示法令**不**鼓励安乐死。相反的，它明确反对安乐死。法令中声明:

> *第17款第1项 – 本法令中的任何条款都不准许造成死亡或加速死亡的行为，这种行为有别于允许死亡过程自然发生的行为。*
>
> *第17款第2项 – 在此声明，本法令中的任何条款都不宽容，准许或认同怂恿自杀的行为，无痛苦致死术或安乐死。*

绝症患者**不能**用预先医疗指示进行自杀。预先医疗指示也不允许医生怂恿任何自杀的企图，这将受到法律惩罚。

## 绝症的证明

## 22. 谁能证明病人已患上绝症?

病人是否已患上绝症必须由三名医生**一致**证明，包括病人在医院的主治医生。其中两名医生必须是专科医生。

## 23. 如果医生小组意见不一致怎么办?

如果第一个由三名医生组成的专门小组就病人是否患上绝症意见不一致，主治医生将对他的诊断进行检讨。如果他仍坚持病人已患上绝症，该病例将提交由卫生部委任的第二个由三名专科医生组成的专门小组决定。

## 24. 如果第二个专门小组对诊断仍不能持一致的看法，预先医疗指示能否生效?

不能。如果第二个专门小组就病人是否患上绝症不能持一致的看法，预先医疗指示**不能**生效。病人的生命将得以维持并获得正常的治疗。

## 25. 我是否须支付由卫生部委任的第二个由三名专科医生组成的专门小组所提供的服务?

不必。

## 预先医疗指示订立之后

### 26. 我是否必须将我已订立预先医疗指示之事通知我的医生或任何人?

您不需向任何人透露此事。不过,在订立预先医疗指示之前最好与您的家人和亲人商量。

### 27. 谁会知道我是否已订立了预先医疗指示?

预先医疗指示的登记册将受保密,当局只会向下列人士透露:

i. 经您书面授权的人
ii. 您的医生,当他有理由相信您已经患上绝症,并且已经没有能力表达您的意愿。

### 28. 已立下预先医疗指示的病人在患上绝症后,医院是否会不进行治疗或让其挨饿?

医生将继续提供临终护理(即为患上绝症的病人进行治疗并减轻他的痛苦)。

即使在预先医疗指示生效后,医院仍将继续提供姑息治疗和药物治疗。

### 29. 什么是临终护理?

临终护理通过预防,评估和治疗痛楚及其他生理,心理与精神方面的问题协助改善绝症患者及其家人的生活素质。

预先医疗指示法令说明临终护理包括:
- 提供合理的医疗程序来舒解痛楚和不适;以及
- 提供适当的食物和水。

### 30. 有什么预防措施能确保在执行预先医疗指示之前我能得到所有必需的治疗?

只要病人没有被证实患上末期绝症,且仍有康复的可能,医生都负有专业责任,确保病人得到所有必需的治疗。即使病人被认为患上不治之症,医生仍有责任提供治疗以减轻病人的痛苦。

这是目前一贯的做法,这种做法会继续下去,无论病人是否有立下预先医疗指示。

## 31. 如果我立下预先医疗指示后又改变主意，我能否取消指示?

预先医疗指示可在任何时候取消，但必须至少有一名证人在场。要取消预先医疗指示应填写**表格3**，这是取消预先医疗指示的标准表格。*（已经订立预先医疗指示的人士将同时收到**表格3**和**表格2**。**表格2**证实其预先医疗指示已经在中央注册局备案）*

又或者，取消预先医疗指示者或其证人可以写封简单的信给预先医疗指示注册官。

### 信内应包含以下资料

(a) 取消预先医疗指示者以及证人的姓名，身份证号码，地址以及住宅与办公室电话号码

(b) 取消预先医疗指示的时间，日期和地点

(c) 如果信是证人写的，必须注明取消预先医疗指示者用以传达他取消该指示意愿的方式（例如，口头，手语）。证人也应阐明取消预先医疗指示者不能亲自写信的原因。

取消预先医疗指示的通知必须尽快寄到中央注册局去。

## 32. 如果我不能书写怎么办?

在这种情况下，预先医疗指示可以通过口头或任何其他您能使用的传达方式予以取消。见证您取消预先医疗指示的证人便有责任用以上所述之表格3或书信的形式呈上取消预先医疗指示的通知。**证人也应阐明取消预先医疗指示者不能亲自填表或写信的原因。**

> *第7款第4项 – 取消医疗指示的见证人有义务将此事通知注册官，不管指示是以书面，口头，或病人所能传达的任何其他方式加以取消的。*

## 如果我没订立预先医疗指示怎么办?

订立预先医疗指示不是强制性的，如果您不想订立，您可以不订立。如果一个绝症患者已陷入昏迷状态，无法表达自己的意愿，同时又没有订立预先医疗指示，现行的做法将继续，那就是医生将征询病人家人的意见，由他们对病人的治疗作决定。

请**记住**，订立预先医疗指示并**不是**到任何医院留医的先决条件。根据法律，它将不会对任何保险单产生影响。

## 预先医疗指示表格详情

1) 这份手册的背面附有一张预先医疗指示的表格（表格1）。您也能够向您的医生（首要证人）索取表格或上网到*http://moh.gov.sg/amdform*下载表格。

2) 您的医生将会为您解释所有的相关措施，手续与何时预先医疗指示将会生效。

3) 他会确保您已过21岁，您是在自愿，完全明白前因后果和神智清醒的情况下订立预先医疗指示的。

   指示神智清醒的例子可包括您能明白您在签什么文件并了解其中所包含的内容；您可以清楚的说出您和亲人的姓名；您知道现在的时间和身在的地点。

4) 当预先医疗指示已有了您和该两名证人的签名，您便可以利用本手册背面附上的预付邮费信封将之寄给预先医疗指示注册局。

5) 当预先医疗指示注册局签收到您的表格时，有关人员会回寄一封确认信（**表格**2）通知您的申请已被承认。

想要知道更多有关订立预先医疗指示的详情，
请拨打卫生部的预先医疗指示热线63259136或上网到卫生部的网站:

*http://www.moh.gov.sg/amd*

# *Arahan Awal Perubatan*

***Kemajuan baru dalam ilmu perubatan dan teknologi mewujudkan pilihan-pilihan baru bagi kedua-dua pesakit dan pihak yang menyediakan jagaan kesihatan. Beberapa pilihan ini pula menimbulkan masalah-masalah baru dari segi etika dan undang-undang.***

***Satu masalahnya ialah teknologi perubatan moden boleh melanjutkan nyawa di tahap-tahap akhir sesuatu penyakit yang membawa maut. Walau bagaimanapun, ia tidak boleh menghentikan proses kematian. Dalam situasi sedemikian, campur tangan perubatan lanjut tidak berkesan dari segi perubatan, dan keputusan perlu dibuat untuk sama ada menarik balik usaha campur tangan perubatan yang sia-sia itu. Ada orang yang menghidap penyakit yang membawa maut dan tidak berdaya lagi untuk meluahkan keinginan mereka pada masa itu mungkin ingin dibebaskan daripada penderitaan lanjut dan dibenarkan meninggal dunia secara semula jadi, dengan aman dan hormat.***

***Undang-undang di Singapura mengizinkan rakyat Singapura yang ingin membuat arahan awal perubatan berbuat demikian. Akta Arahan Awal Perubatan telah diluluskan di Parlimen pada bulan Mei, 1996. Sebelum Akta Arahan Awal Perubatan diperkenalkan, mereka yang ingin mendapat pandangan mengenai rawatan perubatan semasa sakit tenat tidak berpeluang untuk meluahkan keinginan mereka. Oleh itu, keputusan mengenai rawatan perubatan terletak pada doktor dan anggota keluarga mereka.***

## DEFINASI DAN SYARAT-SYARAT

### 1. Apakah Arahan Awal Perubatan?

Arahan Awal Perubatan ialah sebuah dokumen undang-undang yang anda tandatangani dari awal untuk memberitahu doktor yang merawat anda (sekiranya anda menghidap penyakit yang membawa maut dan berada dalam keadaan tidak sedarkan diri) bahawa anda **tidak mahu** sebarang rawatan luar biasa untuk melanjutkan nyawa digunakan bagi memanjangkan usia anda.

Membuat Arahan Awal Perubatan adalah satu keputusan **sukarela**. Terpulang kepada anda sama ada anda mahu membuatnya. Adalah menjadi satu kesalahan jenayah jika ada sesiapa yang memaksa anda untuk membuatnya tanpa kerelaan anda.

### 2. Apakah yang dimaksudkan dengan "penyakit yang membawa maut"?

"**Penyakit yang membawa maut**" ditakrifkan dalam Akta ini sebagai satu keadaan yang tidak boleh disembuhkan yang disebabkan oleh kecederaan atau penyakit di mana tiada harapan yang munasabah untuk pulih sementara atau selama-lamanya. Dalam keadaan demikian, kematian pasti tidak dapat dielakkan walaupun langkah-langkah luar biasa diambil untuk mengekalkan nyawa pesakit. Langkah-langkah luar biasa untuk mengekalkan nyawa ini hanya bertindak menangguhkan ajal bagi pesakit tersebut.

### 3. Apakah maknanya "rawatan luar biasa untuk mengekalkan nyawa"?

"**Rawatan luar biasa untuk mengekalkan nyawa**" adalah sebarang rawatan perubatan yang bertujuan hanya memanjangkan nyawa pesakit-pesakit yang menghidap penyakit yang membawa maut tetapi tidak menyembuhkan penyakit tersebut.

Satu contoh rawatan ini ialah alat pernafasan yang dipasangkan kepada pesakit untuk menolongnya bernafas. Ia hanya memanjangkan nyawa seseorang pesakit yang menghidap penyakit yang membawa maut secara bukan semula jadi.

## MEMBUAT ARAHAN AWAL PERUBATAN

### 4. Siapakah yang boleh membuat Arahan Awal Perubatan? Bagaimanakah saya hendak membuat Arahan Awal Perubatan?

Sesiapa yang berusia 21 tahun ke atas, dan waras boleh membuat Arahan Awal Perubatan. Apa yang anda perlu lakukan ialah mengisi borang Arahan Awal Perubatan, menandatanganinya di hadapan dua orang saksi, dan pulangkannya kepada Pendaftar Arahan Awal Perubatan.

### 5. Di manakah saya boleh dapatkan borang untuk membuat Arahan Awal Perubatan?

Terdapat satu borang (Borang 1) di bahagian belakang buku kecil ini, yang mana anda boleh mendapatkannya daripada klinik-klinik perubatan, poliklinik dan hospital. Anda juga boleh meminta borang tersebut daripada doktor anda apabila anda memutuskan untuk membuat Arahan Awal Perubatan. Selain dari itu, anda juga boleh memuat turun borang Arahan Awal Perubatan di dalam talian *http://www.moh.gov.sg/amdform*

N.B: Bila mencetak borang tersebut, sila cetak di kedua-dua muka surat sekeping kertas.

### 6. Adakah saya perlukan seorang peguam untuk membuat Arahan Awal Perubatan?

Tidak, anda tidak perlukan seorang peguam untuk membuat Arahan Awal Perubatan.

## SAKSI UNTUK ARAHAN AWAL PERUBATAN

### 7. Adakah saya perlu dapatkan nasihat melalui seorang doktor apabila membuat Arahan Awal Perubatan?

Ya. Doktor yang anda temui itu akan bertindak sebagai saksi pertama yang bertanggungjawab untuk memastikan:

(a) Anda tidak dipaksa untuk membuat Arahan Awal Perubatan
(b) Anda berfikiran waras
(c) Anda memahami sifat dan implikasinya apabila membuat Arahan Awal Perubatan

### 8. Siapakah yang boleh menjadi saksi?

Salah seorang daripada dua saksi mestilah seorang doktor, sebaik-baiknya doktor keluarga pesakit tersebut. Saksi yang seorang lagi mestilah berusia sekurang-kurangnya 21 tahun.

Kedua-dua saksi tidak harus mempunyai sebarang kepentingan daripada kematian pesakit. Jika saksi-saksi tersebut adalah saudara mara, selagi mereka tidak mempunyai kepentingan daripada kematian pesakit, mereka akan dibenarkan bertindak sebagai saksi.

Seorang doktor yang mempunyai sebarang sebab untuk membantah sesuatu Arahan Awal Perubatan dan mendaftarkan bantahannya dengan Pendaftar boleh menolak untuk menjadi saksi semasa Arahan Awal Perubatan ditandatangani. Dengan itu, anda boleh mencari doktor lain untuk membuat Arahan Awal Perubatan anda.

### 9. Mestikah kedua-dua saksi hadir semasa Arahan Awal Perubatan dibuat?

Ya, kedua-dua saksi mesti hadir bersama untuk menyaksikan orang yang menandatangani Arahan Awal Perubatan, dan juga untuk menandatangani borang Arahan Awal Perubatan tersebut. Oleh sebab doktor anda adalah saksi pertama anda, jururawatnya boleh menjadi saksi kedua anda atau sesiapa yang sesuai.

### 10. Bolehkah saya mengisi Borang 1 sebelum saya berjumpa doktor?

Anda boleh mengisi butiran diri anda di Borang 1 terlebih dahulu. Walau bagaimanapun, kedua-dua orang saksi anda mesti hadir dan menandatangani borang tersebut bersama-sama anda.

### 11. Berapakah bayarannya untuk membuat Arahan Awal Perubatan?

Untuk membuat Arahan Awal Perubatan, anda mesti dapatkan seorang doktor, sebagai seorang saksi yang diperlukan untuk menerangkan kepada anda mengenai Arahan Awal Perubatan. Anda mungkin perlu membayar khidmat yang diberikan oleh doktor tersebut. Tetapi, borang Arahan Awal Perubatan itu sendiri adalah percuma.

### 12. Bolehkah Arahan Awal Perubatan dibuat sekiranya saya tidak menghidap penyakit yang membawa maut? Haruskah saya memberitahu keluarga saya?

Sebaik-baiknya Arahan Awal Perubatan dibuat semasa tiada tekanan untuk membuatnya - semasa anda sihat. Jika keluarga anda menentangnya, anda boleh mendapatkan kefahaman mereka sebelum membuat Arahan Awal Perubatan. Jika anda tidak mahu memberitahu keluarga anda, anda masih boleh membuat Arahan Awal Perubatan yang akan dirahsiakan, sehingga anda ingin memberitahu mengenainya kepada seseorang.

### 13. Adakah wajib bagi setiap orang dewasa Singapura untuk membuat Arahan Awal Perubatan?

Tidak, ia **TIDAK** wajib. Arahan Awal Perubatan hanya sah apabila ia dibuat secara sukarela. Tiada sesiapa yang boleh memaksa orang lain untuk membuat Arahan Awal Perubatan.

### 14. Bolehkah seorang ibu atau bapa membuat Arahan Awal Perubatan bagi pihak anaknya? Bolehkah seorang ibu atau bapa atau salah seorang adik beradik membuat Arahan Awal Perubatan bagi pihak seorang yang cacat akal?

Tidak seorang pun yang boleh membuat Arahan Awal Perubatan bagi pihak orang lain, sama ada kanak-kanak mahupun orang dewasa.

### 15. Bolehkah anggota keluarga saya menolak atau membantah perlaksanaan Arahan Awal Perubatan saya?

Jika anda telah membuat Arahan Awal Perubatan meskipun keputusan anda dibantah oleh anggota keluarga, para doktor harus menghormati Arahan Awal Perubatan anda. Oleh itu, walaupun tindakan membuat Arahan Awal Perubatan adalah satu keputusan peribadi, kami menggalakkan anda supaya berbincang dengan anggota keluarga anda dan mereka yang disayangi. Ini boleh membantu mereka untuk memahami dan menghormati keinginan anda sekiranya anda sakit tenat.

### 16. Bagaimanakah kita boleh memastikan tidak seorang pun, termasuk mereka yang buta huruf, diperdayakan untuk menandatangani Arahan Awal Perubatan?

Adalah menjadi tugas seorang doktor untuk menerangkan dengan sejelas-jelasnya dan berbincang mengenainya dengan orang yang ingin membuat Arahan Awal Perubatan dan memastikan bahawa dia memahami apa yang ditandatanganinya.

**Memaksa atau memperdayakan orang lain untuk menandatangani Arahan Awal Perubatan adalah satu kesalahan di sisi undang-undang.** Jika didapati bersalah, orang tersebut boleh didenda dan dipenjara dan dibatalkan daripada menjadi pewaris harta.

### 17. Adakah pihak hospital akan mengetahui sekiranya saya membuat Arahan Awal Perubatan?

Kakitangan hospital, termasuk para doktor dan jururawat tidak tahu siapa yang telah membuat Arahan Awal Perubatan kerana ia adalah rahsia, dan mereka **TIDAK** dibenarkan bertanya anda sama ada anda telah membuat Arahan Awal Perubatan. Oleh itu, anda digalakkan untuk memaklumkan kepada kakitangan hospital sekiranya anda telah membuat Arahan Awal Perubatan. Walau bagaimanapun, jika doktor yang merawat anda mempunyai sebab untuk mempercayai bahawa anda menghidap penyakit yang boleh membawa maut dan tidak dapat meluahkan keinginan anda kepadanya, dia akan memeriksa dengan Pendaftar Arahan Awal Perubatan sama ada anda telah membuat Arahan Awal Perubatan.

### 18. Bolehkah saya membuat Arahan Awal Perubatan semasa berada di luar negara?

Asalkan saksi pertama anda adalah seorang doktor yang masih berdaftar dengan Singapore Medical Council (SMC), dan Arahan Awal Perubatan difailkan dengan Pendaftar Arahan Awal Perubatan di Singapura, Arahan Awal Perubatan itu dikira sah.

### 19. Bolehkah Arahan Awal Perubatan dijalankan semasa saya berada di luar negara?

Apabila anda berada di luar negara, status sah Arahan Awal Perubatan akan bergantung kepada undang-undang tempatan bagi negara di mana anda berada. Kerana itulah Arahan Awal Perubatan biasanya dipraktikkan/dilaksanakan apabila anda berada di Singapura.

## ARAHAN AWAL PERUBATAN BERBANDING DENGAN EUTANASIA

### 20. Adakah Arahan Awal Perubatan itu sejenis eutanasia atau pembunuhan belas kasihan?

Tidak, Arahan Awal Perubatan **bukan** eutanasia atau pembunuhan belas kasihan. Eutanasia/pembunuhan belas kasihan ialah perbuatan yang dilakukan dengan sengaja untuk menamatkan nyawa seseorang yang menghidap penyakit yang tidak boleh disembuhkan dan yang sangat menyakitkan dengan cara luar biasa, seperti penyuntikan kimia beracun.

Arahan Awal Perubatan bertindak sebagai arahan awal kepada doktor anda supaya tidak melanjutkan nyawa anda dengan rawatan luar biasa bagi mengekalkan nyawa dan membiarkan anda menemui ajal melalui proses nazak secara semula jadi sekiranya anda menghidap penyakit yang membawa maut dan apabila anda tidak sedarkan diri.

### 21. Adakah Akta Arahan Awal Perubatan menggalakkan eutanasia?

Tidak, Akta Arahan Awal Perubatan **TIDAK** menggalakkan eutanasia. Sebaliknya, Akta ini terang-terang menentang eutanasia. Akta ini menyatakan:

> ***Nazak Seksyen 17(1) - Tidak ada sesuatu dalam Akta ini yang akan memberi kuasa untuk sebarang tindakan yang menyebabkan atau mempercepatkan kematian yang berbeza daripada tindakan yang mengizinkan proses nazak berlaku secara semula jadi.***
>
> ***Seksyen 17(2) - Dengan ini mengisytiharkan bahawa tidak ada sesuatu pun dalam Akta ini yang akan membiarkan, memberi kuasa atau membenarkan perbuatan subahat dalam hal bunuh diri, pembunuhan belas kasihan terhadap pesakit atau eutanasia.***

Seorang pesakit yang menghidap penyakit yang membawa maut **TIDAK** boleh menggunakan Arahan Awal Perubatan untuk membunuh diri, begitu juga Arahan Awal Perubatan tidak membenarkan para doktor membantu sebarang percubaan membunuh diri. Hukuman undang-undang akan dikenakan ke atas sesiapa yang didapati melakukan kesalahan ini.

## PENGESAHAN PENYAKIT YANG MEMBAWA MAUT

### 22. Siapakah yang boleh mengesahkan seseorang itu menghidap penyakit yang membawa maut?

Tiga orang doktor termasuk doktor hospital pesakit itu mestilah mengesahkan dengan sebulat suara bahawa penyakit yang dihidapinya membawa maut. Dua daripada doktor tersebut mestilah doktor pakar.

### 23. Apakah akan jadi jika panel doktor tidak mencapai keputusan sebulat suara?

Jika panel pertama yang terdiri daripada 3 orang doktor tidak dapat mencapai persetujuan sebulat suara bahawa pesakit itu menghidap penyakit yang membawa maut, doktor yang bertanggungjawab akan mengkaji semula diagnosisnya. Jika dia masih berpendapat bahawa pesakit itu menghidapi penyakit yang membawa maut, perkara itu akan dirujuk kepada panel kedua yang terdiri daripada 3 orang doktor pakar, yang akan dilantik oleh Kementerian Kesihatan.

### 24. Bolehkah Arahan Awal Perubatan berkuatkuasa jika panel doktor yang kedua juga tidak mencapai persetujuan tentang diagnosis itu?

Tidak, Arahan Awal Perubatan **tidak boleh** berkuatkuasa jika panel kedua tidak mencapai persetujuan dengan sebulat suara bahawa pesakit itu menghidap penyakit yang membawa maut. Nyawa pesakit akan terus dikekalkan dan dia akan diberi rawatan perubatan seperti biasa.

### 25. Perlukah saya membayar bagi khidmat panel 3 doktor pakar kedua yang dilantik oleh Kementerian Kesihatan?

Anda tidak perlu membayar bagi khidmat mereka.

## APA YANG BERLAKU SETELAH ARAHAN AWAL PERUBATAN DIBUAT?

### 26. Perlukah saya memberitahu doktor saya atau sesiapa sahaja mengenai Arahan Awal Perubatan yang dibuat oleh saya?

Anda tidak perlu mendedahkan kepada sesiapa mengenai Arahan Awal Perubatan yang dibuat oleh anda. Walau bagaimanapun, dinasihatkan supaya anda membincangkannya dengan keluarga anda dan mereka yang disayangi sebelum membuat Arahan Awal Perubatan.

### 27. Siapakah yang akan mengetahui sama ada saya telah membuat Arahan Awal Perubatan?

Daftar Arahan Awal Perubatan adalah rahsia dan hanya boleh didedahkan kepada:

i. individu-individu yang diberi kuasa oleh anda secara bertulis;
ii. doktor anda apabila dia mempunyai sebab untuk mempercayai bahawa anda menghidap penyakit yang membawa maut dan tidak berupaya untuk memberitahu keinginan anda kepadanya.

### 28. Jika seorang pesakit yang membuat Arahan Awal Perubatan menghidap penyakit yang membawa maut, adakah ia akan dibiarkan tanpa rawatan perubatan atau dibiarkan kelaparan?

Para doktor akan terus memberi jagaan pelegaan (iaitu merawat dan melegakan kesakitan dan penderitaan pesakit yang menghidap penyakit yang membawa maut).

Walaupun Arahan Awal Perubatan telah berkuatkuasa, jagaan pelegaan dan perubatan akan terus disediakan.

### 29. Apakah dia jagaan pelegaan?

Jagaan pelegaan membantu memperbaiki mutu kehidupan pesakit yang menghidapi penyakit yang membawa maut dan anggota keluarga mereka melalui pencegahan, penilaian, dan rawatan kesakitan dan masalah-masalah fizikal, psikologi, dan kerohanian yang lain.

Akta Arahan Awal Perubatan menetapkan yang jagaan pelegaan adalah termasuk:
- pemberian prosedur-prosedur perubatan yang berpatutan bagi melegakan kesakitan, penderitaan atau ketidakselesaan; dan
- pemberian makanan dan air yang berpatutan.

### 30. Apakah langkah-langkah perlindungan yang diambil untuk memastikan bahawa saya diberi rawatan yang diperlukan sebelum Arahan Awal Perubatan dilaksanakan?

Ia adalah satu tanggungjawab profesional setiap doktor untuk memastikan bahawa semua rawatan yang diperlukan diberi kepada pesakit selagi pesakit itu tidak disahkan menghidap penyakit yang membawa maut dan terdapat kemungkinan bahawa ia akan pulih. Walaupun pesakit itu dianggap menghidap penyakit yang membawa maut, seseorang doktor itu mempunyai tanggungjawab untuk merawatnya bagi meringankan sakit dan penderitaannya.

Ini adalah amalan semasa, dan akan diteruskan sama ada Arahan Awal Perubatan telah dibuat oleh pesakit tersebut atau tidak.

## MEMBATALKAN ARAHAN AWAL PERUBATAN

### 31. Jika saya menukar fikiran mengenai Arahan Awal Perubatan yang telah saya buat lebih awal, bolehkah saya batalkannya?

Arahan Awal Perubatan boleh dibatalkan pada bila-bila masa di hadapan sekurang-kurangnya seorang saksi. Orang yang melakukan pembatalan boleh berbuat demikian dengan mengisi **Borang 3**, yang merupakan borang rasmi yang disediakan bagi pembatalan Arahan Awal Perubatan. *(Mereka yang telah membuat Arahan Awal Perubatan akan menerima **Borang 3** bersama dengan **Borang 2**, yang mengesahkan bahawa Arahan Awal Perubatan mereka telah didaftarkan oleh Pejabat Pendaftaran Arahan Awal Perubatan)*

Selain dari itu, orang tersebut atau saksinya boleh menulis sepucuk surat yang mudah kepada Pendaftar Arahan Awal Perubatan.

**Surat itu hendaklah mengandungi maklumat berikut:**

(a) nama dan nombor Kad Pengenalan kedua-dua orang yang membatalkan Arahan Awal Perubatan dan saksinya, berserta alamat rumah dan nombor telefon rumah dan pejabat mereka

(b) masa, tarikh dan tempat di mana pembatalan itu dilakukan

(c) jika surat itu ditulis oleh saksi, cara perhubungan yang seseorang itu gunakan untuk menyampaikan niatnya bagi memansuhkan Arahan Awal Perubatan (contohnya, secara lisan, bahasa isyarat). Saksi tersebut harus menyatakan sebab mengapa orang yang membatalkan Arahan Awal Perubatan tidak boleh melakukannya sendiri.

Pembatalan hendaklah dihantar ke Pejabat Pendaftaran Arahan Awal Perubatan secepat mungkin.

### 32. Bagaimana sekiranya keadaan saya ini tidak mengizinkan saya untuk menulis?

Dalam keadaan ini, Arahan Awal Perubatan boleh dibatalkan secara lisan atau dengan sebarang cara lain yang membolehkan anda berkomunikasi. Adalah menjadi tanggungjawab saksi kepada pembatalan tersebut untuk menghantar notis pembatalan dengan menggunakan Borang 3 atau surat yang diterangkan di atas. **Saksi tersebut harus menyatakan sebab mengapa orang yang membatalkan Arahan Awal Perubatan tidak boleh melakukannya sendiri.**

***Seksyen 17(4) - Adalah menjadi tugas orang "yang telah menyaksikan pembatalan, sama ada yang dibuat secara bertulis, lisan atau dengan sebarang cara lain yang mana pesakit dapat berkomunikasi, untuk memaklumkan kepada Pendaftar mengenai pembatalan itu.***

## BAGAIMANA JIKA SAYA TIDAK MEMPUNYAI ARAHAN AWAL PERUBATAN?

Arahan Awal Perubatan tidak wajib dibuat. Sekiranya anda tidak ingin berbuat demikian. Bagi pesakit yang menghidap penyakit yang membawa maut yang belum membuat Arahan Awal Perubatan, berada dalam keadaan tidak sedarkan diri dan tidak berdaya untuk memberitahu keinginannya, amalan semasa hendaklah diteruskan. Doktor akan berbincang dengan anggota keluarga pesakit untuk memutuskan rawatan bagi pesakit.

**Ingatlah**, membuat Arahan Awal Perubatan **bukan** merupakan syarat untuk memasuki sebarang hospital. Mengikut undang-undang, Arahan Awal Perubatan tidak menjejas sebarang polisi insuran.

## RINGKASAN UNTUK BORANG ARAHAN AWAL PERUBATAN

1) Borang untuk membuat Arahan Awal Perubatan (Borang 1) ada terdapat di belakang buku kecil ini. Anda juga boleh mendapatkannya daripada doktor anda yang mesti menjadi saksi pertama anda. Borang itu juga boleh dimuat turun dari laman web MOH: *http://moh.gov.sg/amdform*

2) Doktor anda akan menjelaskan tentang perlindungan-perlindungan, prosedur-prosedur dan bila Arahan Awal Perubatan dilaksanakan.

3) Dia akan memastikan yang anda berusia lebih 21 tahun, anda menandatangani secara sukarela, anda faham akibat-akibat apabila menandatanganinya dan anda berfikiran waras.

   Tanda-tanda berfikiran waras adalah apabila anda memahami apa yang anda tandatangani dan implikasi-implikasinya, juga anda boleh nyatakan nama anda dan ahli keluarga anda dan anda tahu masa dan alamat pada masa itu.

4) Apabila borang Arahan Awal Perubatan telah ditandatangani oleh anda dan dua orang saksi, anda boleh menghantarnya ke Pejabat Pendaftaran Arahan Awal Perubatan dengan menggunakan sampul surat berbayar yang dilampirkan di belakang buku kecil ini.

5) Apabila Pejabat Pendaftaran Arahan Awal Perubatan telah menerima borang anda, ia akan menghantar surat penerimaan **(Borang 2)** kepada anda untuk mengesahkan yang anda telah didaftarkan dalam Pendaftaran Arahan Awal Perubatan.

Untuk mendapatkan maklumat lanjut mengenai Arahan Awal Perubatan
Hubungi talian hotline Kementerian Kesihatan di 6325 9136
atau kunjungi laman web MOH di

*http://www.moh.gov.sg/amd*

# மருத்துவ முன் உத்தரவு

*மருத்துவ அறிவியலிலும் தொழில்நுட்பத்திலும் ஏற்பட்டுள்ள புதிய முன்னேற்றங்கள் நோயாளிகளுக்கும் சுகாதார சேவை வழங்குவோருக்கும் புதிய விருப்பத் தெரிவு உரிமைகளை அளித்துள்ளன. இவற்றில் சில அறநெறி, சட்ட ரீதியில் புதிய பிரச்சனைகளை ஏற்படுத்தியுள்ளன.*

*மரணத்தை ஏற்படுத்தும் ஒரு நோயின் இறுதிக் கட்டத்தின்போது நவீன மருத்துவ தொழில்நுட்பம் மூலம் ஓர் உயிரைத் தொழில்நுட்ப ரீதியில் நீடிக்கச் செய்ய முடியும் என்பது ஒரு பிரச்சனை. இறப்பை மருத்துவ தொழில்நுட்பம் ஒருபோதும் தடுத்து நிறுத்த முடியாது. மருத்துவ தலையீட்டின் மூலம் பலன் கிடைக்காத இத்தகைய சூழ்நிலைகளில் பயனற்ற மருத்துவத் தலையீட்டை மீட்டுக்கொள்ள முடிவு எடுக்கப்பட வேண்டும். மரணத்தை ஏற்படுத்தும் நோய் முற்றிய நோயாளிகளில் சிலர், மேலும் அவதியுறாமல் அமைதியுடனும் மதிப்புடனும் இயல்பாக இறக்க அனுமதிக்கப்படுவதை விரும்பக்கூடும். ஆனால், அவர்கள் தம் விருப்பத்தை அந்த நேரத்தில் வெளிப்படுத்த இயலாத நிலையில் இருக்கலாம்.*

*மருத்துவ முன் உத்தரவு பிறப்பிக்க விரும்பும் சிங்கப்பூரர்கள் இவ்வாறு செய்வதற்குச் சிங்கப்பூரிலுள்ள சட்டம் அனுமதி அளிக்கறது. மருத்துவ முன் உத்தரவு, 1996 மே மாதத்தில் நாடாளுமன்றத்தில் சட்டம் ஆனது. மருத்துவ முன் உத்தரவு சட்டத்திற்கு முன்பு, ஒருவர் தனக்கு நோய் முற்றிய நிலையில் தனக்கு எத்தகைய சிகிச்சை அளிக்கப்படவேண்டும் என்பதை முன்கூட்டியே தெரிவிக்க இயலாத நிலை இருந்தது. எனவே எத்தகைய சகிச்சை அளிக்கப்படவேண்டும் என்பதை அவர்களின் மருத்துவரும், குடும்ப உறுப்பினர்களுமே தீர்மானித்தனர்.*

## விதிகளும், விளக்கங்களும்

### 1. மருத்துவ முன் உத்தரவு என்பது என்ன?

(மரணத்தை ஏற்படுத்தும் நோய் முற்றிய நிலையில் நீங்கள் இருப்பதுடன் உங்கள் சுய நினைவை இழக்கவும் நேரிட்டால்), சிறப்பு உயிர்காப்பு சிகிச்சை எதுவும் **செய்யக்கூடாது** எனும் உங்கள் விருப்பத்தை உங்களுக்கு சிகிச்சை அளித்து வரும் மருத்துவரிடம் முன்கூட்டியே தெரிவிக்கிற, நீங்கள் கையொப்பம் இட்ட, சட்ட பூர்வமான பத்திரம் இந்த மருத்துவ முன் உத்தரவு.

ஒரு மருத்துவ முன் உத்தரவை சமர்ப்பிப்பது, உங்களின் **தன்னிச்சையான** முடிவே. அவ்வாறு செய்ய விரும்புவது முற்றிலும் உங்கள் விருப்பம். யாரேனும் உங்களை வற்புறுத்தி, உங்கள் விருப்பத்திற்கு மாறாக கோரிக்கைகளை சமர்ப்பிக்கச் செய்தால், அது சட்டப்படி குற்றமாகும்.

### 2. "மரணத்தை ஏற்படுத்தும் நோய்" என்றால் என்ன?

காயம் அல்லது நோயினால் ஏற்படும் குணப்படுத்தமுடியாத ஒரு பிணியிலிருந்து தற்காலிகமாக அல்லது நிரந்தரமாக மீள வாய்ப்பு இல்லை என்றால் அது **"மரணத்தை ஏற்படுத்தும் நோய்"**. இத்தகைய நிலையில் சிறப்பு உயிர்காப்பு சிகிச்சை போன்ற நடவடிக்கைகளை மேற்கொண்டாலும் மரணம் ஏற்படுவது நிச்சயம். இத்தகை நடவடிக்கைகள் நோயாளியின் மரணத்தைத் சில நாட்கள் தள்ளி வைக்க மட்டுமே உதவும்.

### 3. "சிறப்பு உயிர்காப்பு சிகிச்சை" என்றால் என்ன?

மரணத்தை ஏற்படுத்தும் நோயின் இறுதிக் கட்டத்தில் இருக்கும் நோயாளியின் மரணத்தைத் தடுத்து நிறுத்த முடியாத நிலையில் அதைத் தள்ளி வைக்க உதவுகிற மருத்துவ சிகிச்சையே **"சிறப்பு உயிர்காப்பு சிகிச்சை"**. இந்த சிகிச்சை நோயைக் குணப்படுத்துவது இல்லை.

நோயாளி சுவாசிக்க உதவுவதற்காக அவர் உடலில் இணைக்கப்படுகிற சுவாசக் கருவி இதற்கு ஓர் எடுத்துக்காட்டு. இந்நோயாளிகளில் சிலரின் உயிரை நீட்டிக்க, சுவாசக் கருவி உதவுகிறது.

## மருத்துவ முன் உத்தரவை பிறப்பித்தல்

### 4. மருத்துவ முன் உத்தரவை யார் பிறப்பிக்க முடியும்? இந்த உத்தரவை நான் எப்படி பிறப்பிக்க வேண்டும்?

21 வயதான அல்லது அதற்கு மேற்பட்ட ஒருவர் சரியான மனநிலையில் இருந்தால், மருத்துவ முன் உத்தரவை பிறப்பிக்க முடியும்.நீங்கள் செய்ய வேண்டியதெல்லாம், குறிப்பிட்ட மருத்துவ முன் உத்தரவு படிவத்தை நிரப்பி, சாட்சிகள் இருவரின் முன்நிலையில் அதில் கையொப்பம் இட்டபின் மருத்துவ முன் உத்தரவு பதிவாளரிடம் கொடுக்கவேண்டும்.

### 5. மருத்துவ முன் உத்தரவை சமர்ப்பிப்பதற்கான படிவங்களை நான் எங்கு பெற்றுக்கொள்ளலாம்?

இந்த கையேட்டின் இறுதியில், ஒரு படிவம் (படிவம் 1) இணைக்கப்பட்டிருக்கிறது. மருத்துவ மருந்தகங்களிலும், பலதுறை மருந்தகங்களிலும், பிற மருத்துவமனைகளிலும் இந்த கையேடுகள் கிடைக்கும். நீங்கள் மருத்துவ முன் உத்தரவை சமர்ப்பிக்க முடிவு செய்து விட்டால், உங்கள் மருத்துவரிடமும் அதற்கான படிவத்தைப் பெற்றுக்கொள்ளலாம்.

மேலும், *http:/www. moh. gov. sg/amdform* என்ற இணையத்தளத்தில் இருந்தும் நீங்கள் படிவங்களை மின்னியல் இறக்கம் செய்து கொள்ளலாம். பின்குறிப்பு: படிவங்களை அச்சிடும்போது, தயவு செய்து ஒரு தாளின் இரு பக்கங்களிலும் அச்சிடுங்கள்.

### 6. மருத்துவ முன் உத்தரவை சமர்ப்பிக்க நான் வழக்குறைஞரை நாட வேண்டுமா?

வேண்டாம். மருத்துவ முன் உத்தரவை பூர்த்தி செய்து சமர்ப்பிக்க உங்களுக்கு ஒரு வழக்குறைஞர் தேவை இல்லை.

## மருத்துவ முன் உத்தரவு சாட்சி

### 7. மருத்துவ முன் உத்தரவை சமர்பிக்கும்போது, நான் ஒரு மருத்துவரை அணுக வேண்டுமா?

ஆமாம். நீங்கள் அணுகும் மருத்துவர் உங்கள் முதல் சாட்சியாக இருப்பார். அவரின் பொறுப்புகள்:

(a) மருத்துவ முன் உத்தரவை சமர்ப்பிப்பதில், உங்களை யாரும் வற்புறுத்தவில்லை என்பதை உறுதி செய்வது.
(b) நீங்கள் தெளிவான சிந்தனையில் இருக்கிறீர்களா என்பதை உறுதி செய்வது.
(c) மருத்துவ முன் உத்தரவை சமர்ப்பிர்பதன் காரணங்கள்,விளைவுகள், அனைத்தையும் நீங்கள் நன்கு புரிந்துகொண்டிருக்கிறீர்களா என்பதை உறுதி செய்வது.

### 8. யார் சாட்சியாக இருக்க முடியும்?

இரு சாட்சிகளில் ஒருவர் மருத்துவராக இருக்க வேண்டும்; அவர் நோயாளியின் குடும்ப மருத்துவராக இருப்பது நல்லது. இன்னொரு சாட்சி குறைந்தது 21 வயதினராக இருக்க வேண்டும்.

இரு சாட்சிகளும் உங்கள் மரணத்தினால் பயன் பெறுபவர்களாக இருக்க கூடாது. நோயாளியின் உறவினர்கள், நோயாளியின் இறப்பினால் தாங்கள் எவ்விதத்திலும் பயன் பெறாதவர்களாக இருக்கும் பட்சத்தில் அவர்கள் சாட்சியாக இருக்க முடியும்.

மருத்துவ முன் உத்தரவை மருத்துவர் ஏதேனும் காரணத்தினால் எதிர்த்தால் அவர் தம் எதிர்ப்பைப் பதிவாளரிடம் தெரிவித்த பின் சாட்சியாக இருக்க மறுக்கலாம். பிறகு, மருத்துவ முன் உத்தரவுக்கு ஏற்பாடு செய்வதற்கு மற்றொரு மருத்துவரை நீங்கள் நாடலாம்.

**9. மருத்துவ முன் உத்தரவில் கையெழுத்திடும்போது இரண்டு சாட்சிகள் உடன் இருக்க வேண்டுமா?**

ஆம். மருத்துவ முன் உத்திரவில் கையெழுத்திடுபவரின் அடையாளத்தை உறுதிபடுத்தவும் உத்தரவுப் படிவத்தில் கையெழுத்திடவும் இரண்டு சாட்சிகள் உடன் இருக்க வேண்டும். முதல் சாட்சி உங்கள் மருத்துவராக இருப்பதால், இரண்டாவது சாட்சி அவரின் தாதியாகவோ அல்லது தகுந்த வேறு நபராகவோ இருக்கலாம்.

**10. மருத்துவரை நாடுவதற்கு முன்னதாக நான் படிவம் ஒன்றை பூர்த்தி செய்யலாமா?**

நீங்கள் படிவம் ஒன்றை முதலில் பூர்த்தி செய்யலாம். இருப்பினும் இரண்டு சாட்சிகளும் நீங்களும் ஒரே நேரத்தில் அந்த படிவத்தில் கையெழுத்திட வேண்டும்.

**11. மருத்துவ முன் உத்தரவு நிறைவேற்றப்படுவதற்கு என்ன செலவாகும்?**

மருத்துவ முன் உத்தரவை சமர்ப்பிப்பதற்கு நீங்கள் ஒரு மருத்துவரை நாட வேண்டும். உங்களின் சாட்சியாக பொறுப்பு வகிக்கும் அவர், மருத்துவ முன் உத்தரவு பற்றிய தகவல்களை உங்களுக்கு விளக்கவேண்டும். அவர் வழங்கும் சேவைக்கு நீங்கள் கட்டணம் செலுத்தவேண்டியிருக்கலாம். இருப்பினும், மருத்துவ முன் உத்தரவு படிவம் உங்களுக்கு இலவசமாகவே கிடைக்கும்.

**12. எனக்கு இறப்பை ஏற்படுத்தும் நோய் இல்லையெனில் மருத்துவ முன் உத்தரவுக்கு நான் ஆவன செய்யலாமா?என் குடும்பத்துக்கு நான் தெரியப்படுத்தவேண்டுமா?**

நீங்கள் மருத்துவ முன் உத்தரவுக்கு ஆவன செய்யவேண்டிய நெருக்குதலுக்கு உள்ளாகாத வேளையில்-அதாவது உடல் நலத்துடன் இருக்கையில் -ஆவன செய்வதே சிறந்தது. உங்கள் குடும்பம் அதற்கு எதிர்ப்பு தெரிவித்தால் ஆவன செய்வதற்கு முன் அவர்களின் புரிந்துணர்வைப் பெற நீங்கள் முயலலாம். உங்கள் குடும்பத்தினரிடம் தெரிவிக்க விரும்பாவிட்டாலும் மருத்துவ மன் உத்தரவுக்கு நீங்கள் ஆவன செய்யலாம். இது குறித்து நீங்கள் யாருக்கும் தெரிவிக்கும்வரை இந்த ஏற்பாடு இரகசியமாக இருக்கும்.

**13. சிங்கப்பூரில்,வயது வந்த ஒவ்வொருவரும் மருத்துவ முன் உத்தரவுக்கு கட்டாயமாக ஏற்பாடு செய்ய வேண்டுமா?**

வேண்டாம். இது கட்டாயம் **அல்ல**. தன்னிச்சையாக முடிவு செய்து, சமர்ப்பிக்கப்படும் மருத்துவ முன் உத்தரவு படிவங்களே செல்லத்தக்கதாகும். மற்றவர்கள் யாரும் உங்களை இதற்கு வற்புறுத்த முடியாது.

**14. பெற்றோரில் ஒருவர் தன் பிள்ளை சார்பில் மருத்துவ முன் உத்தரவுக்கு ஆவன செய்யலாமா? மனநிலை சரியில்லாத ஒருவரின் சார்பில் அவருடைய தாயோ தந்தையோ உடன்பிறந்தவரோ மருத்துவ முன் உத்தரவுக்கு ஆவன செய்யலாமா?**

பிள்ளையோ, வயது வந்தவரோ, இன்னொருவர் சார்பில் வேறு எவருமோ மருத்துவ முன் உத்தரவைப் பிறப்பிக்க முடியாது.

**15. மருத்துவ முன் உத்தரவுக்கு நான் ஏற்பாடு செய்வதை என் குடும்ப உறுப்பினர்க எதிர்த்தால் எப்படி?**

உங்கள் குடும்ப உறுப்பினர்களின் எதிர்ப்பைப் பொருட்படுத்தாமல் நீங்கள் மருத்துவ முன் உத்தரவில் கையெழுத்து இட்டிருந்தாலும் மருத்துவர்கள் அதனை மதிக்க வேண்டும். மருத்துவ முன் உத்தரவை சமர்ப்பிப்பது முற்றிலும் உங்கள் தனிப்பட்ட முடிவாக இருக்க வேண்டும் என்றாலும், நீங்கள் அதை உங்கள் குடும்பத்தினரோடும், அன்புக்குறியவர்களோடும் கலந்து ஆலோசிப்பது சிறந்தது. நீங்கள் மரணத்தை எதிர்நோக்கும் அளவிற்கு நோய்வாய்ப்படும் சூழ்நிலைகளில், உங்கள் விருப்பங்களை புரிந்துகொண்டு, மதித்து நடக்க இது அவர்களுக்கு உதவியாக இருக்கும்.

## 16. மருத்துவ முன் உத்தரவில் படிப்பறிவில்லாதவர் உள்ளிட்ட எவரும் தெரியாமல் கையெழுத்திட நேரிடாது என்பதை எப்படி உறுதிப்படுத்திக்கொள்ள முடியும்?

அவர் எதில் கையெழுத்திடுகிறார் என்பதை நன்கு உணரும் வகையில் அவருக்கு அதைப் பற்றி முழு விளக்கம் அளிப்பதும் அவருடன் கலந்து பேசுவதும் மருத்துவரின் கடமையாகும்.

**மருத்துவ முன் உத்தரவில் கையெழுத்திட ஒருவரைக் கட்டாயப்படுத்துவதோ தவறான வழி காட்டுவதோ சட்டப்படி குற்றம்.குற்றம் மெய்பிக்கப்பட்டால் அபராதமும் சிறைவாசமும் விதிக்கப்படும்.** மேலும் பயன்பெறுபவராக இருப்பின் பயன் பெறும் தகுதியை இழக்கவும் நேரிடும்.

## 17. மருத்துவ முன் உத்தரவை நான் பிறப்பித்திருப்பதை மருத்துவமனை அறியுமா?

மருத்துவ முன் உத்திரவு ரகசியமாக இருப்பதால் அதை யார் பிறப்பித்து இருக்கிறார் என்பது மருத்துவர்கள், தாதியர்கள் உட்பட மருத்துவமனை ஊழியர்களில் யாருக்கும் தெரியாது. நீங்கள் இந்த உத்தரவைப் பிறப்பித்து இருக்கிருக்கிறீர்களா என்பதைக் கேட்பதற்கும் அவர்களுக்கு அனுமதி **இல்லை** எனினும், நீங்கள் மருத்துவ முன் உத்தரவைப் பிறப்பித்திருந்தால் அதை மருத்துவ ஊழியரிடம் தெரிவிப்பது நல்லது. எனினும் உங்களை கவனிக்கும் மருத்துவர் நீங்கள் நோய் முற்றிய நிலையில் இருப்பதாகவும், உங்களால் உங்கள் விருப்பத்தை அவரிடம் தெரிவிக்க இயலாதவர் என்று அவர் கருதினால், அவர் மருத்துவ முன் உத்தரவு பதிவகத்தாரிடம் நீங்கள் மருத்துவ முன் உத்தரவை பிறப்பித்திருக்கிறீர்களா என்று விசாரிக்கலாம்.

## 18. நான் வெளிநாட்டில் இருக்கும்போது மருத்துவ முன் உத்தரவை சமர்பிக்கலாமா?

உங்கள் முதல் சாட்சி, சிங்கப்பூர் மருத்துவ கழகத்தின் அங்கீகாரம் பெற்ற ஒரு மருத்துவராக இருந்தாலும், உங்கள் மருத்துவ முன் உத்தரவு படிவத்தை, நீங்கள் சிங்கப்பூர் எஸ்டி பதிவகத்திடம் சமர்ப்பித்து இருந்தாலும், உங்கள் மருத்துவ முன் உத்தரவு செல்லத்தக்கதாகக் கருதப்படும்.

## 19. நான் வெனிநாட்டில் இருக்கும்பொது மருத்துவ முன் உத்தரவை நடைமுறைப்படுத்த முடியுமா?

நான் வெளி நாட்டில் இருக்கும்போது மருத்துவ முன் உத்தரவை சட்டப்பூர்வ நடைமுறைகள் அந்த நாட்டு சட்டத்திற்கேற்ப அமையும். ஆகவே, உங்கள் மருத்துவ முன் உத்தரவை நீங்கள் சிங்கப்பூரில் இருக்கும்போது மட்டுமே நடைமுறைபடுத்த முடியும்.

# மருத்துவ முன் உத்தரவும் கருணைக்கொலையும்

## 20. மருத்துவ முன் உத்தரவு என்பது கருணைக் கொலையா?

இல்லை. மருத்துவ முன் உத்தரவு என்பது கருணைக் கொலை **அல்ல**. கொல்லக்கூடிய இரசாயனங்களை உடலுக்குள் செலுத்துதல் போன்ற செயற்கை வழிகள் மூலம், வலி தருகிற தீராத நோயினால் அவதியுறும் நோயாளியின் உயிரை வேண்டுமென்றே போக்குவது கருணைக் கொலை.

மரணத்தை ஏற்படுத்தும் நோய் முற்றிய நிலையில் நீங்கள் இருந்துஉங்கள் சுயநினைவை இழக்கும்போது நீங்கள் விரும்பும் மருத்துவச் சிகிச்சை பற்றி உங்கள் மருத்துவர்களுக்கு முன்கூட்டியே உத்தரவு பிறப்பிக்கவே இந்த மருத்துவ முன் உத்தரவு செயல்படுகிறது. சிறப்பு உயிர்க்காப்பு சிகிச்சை மூலம் உங்கள் உயிரை நீட்டிக்க வேண்டாம் என்றும் நோய் முற்றிய நிலையில் மரணம் இயற்கையாக நிகழவும் அனுமதிக்கிறது.

## 21. மருத்துவ முன் உத்திரவு கருணைக்கொலை ஊக்குவிக்கிறதா?

இல்லை.மருத்து முன் உத்திரவு கருணைக் கொலையை **ஊக்குவிக்கவில்லை**. மாறாக முன் உத்தரவுச் சட்டம் வெளிப்படையாகவும் திட்டவட்டமாகவும் கருணைக் கொலைக்கு எதிரானது. இந்தச் சட்டம் கீழ்காணும் பிரிவுகளின்கீழ் கூறுவதாவது:

> *பிரிவு **17(1)** இயற்கையாக மரணம் ஏற்படுத்துவதற்கு எதிரான முறையில் மரணத்தை விளைவிக்கும் அல்லது விரைவுபடுத்தும் எந்தச் செயலையும் இச்சட்டம் அனுமதிக்காது.*
>
> *பிரிவு **17(2)** தற்கொலை, கருணைக்கொலை போன்றவற்றை ஆதரிக்கவோ, ஒப்புதல் அளிக்கவோ, அங்கீகரிக்கவோ இச்சட்டத்தில் விதி எதுவுமில்லை என்று இதன் மூலம் தெரிவிக்கப்படுகிறது.*

மரணக் கட்டத்தில் இருக்கும் நோயாளி தற்கொலை செய்வதற்கு மருத்துவ முன் உத்தரவைப் பயன்படுத்த முடியாது. இத்தகைய தற்கொலை முயற்சியில் மருத்துவர்கள் உதவி செய்வதையும் இந்த உத்தரவு அனுமதிக்காது. இத்தகைய செயல் சட்டத்தின்கீழ் தண்டனைக்கு உரியது.

## மரணத்தை ஏற்படுத்தும் நோய் ஒருவருக்கு உண்டா என்று உறுதிப்படுத்துதல்.

## 22. மரணத்தை ஏற்படுத்தும் நோய் முற்றிய நிலையில் நோயாளி இருக்கிறார் என்று யார் உறுதி கூறலாம்?

நோயாளியை மருத்துவமனையில் கவனிக்கும் மருத்துவர் உட்பட மூன்று மருத்துவர்கள், மரணத்தை ஏற்படுத்தும் நோய் முற்றிய நிலையில் நோயாளி இருக்கிறார் என்பதை ஒரு மனதாக உறுதிபடுத்த வேண்டும். அந்த மருத்துவர்களில் இருவர் நிபுணர்களாக இருக்க வேண்டும்.

## 23. மருத்துவ குழு ஒருமித்த முடிவு எடுக்க இயலாவிட்டால் எப்படி?

மரணக்கட்டத்தில் நோயாளி இருக்கிறார் என்று மூன்று மருத்துவர்களும் ஒருமித்த முடிவுக்கு வர முடியாவிட்டால், பொறுப்பிலுள்ள மருத்துவர் நோயாளியின் நிலையை மீண்டும் பரிசீலனை செய்வார். நோயாளி மரணக்கட்டத்தில் இருக்கிறார் என்று மருத்துவர் இன்னமும் கருதினால் சுகாதார அமைச்சு நியமிக்கும் மூன்று பேர் அடங்கிய இரண்டாவது மருத்துவ நிபுணர் குழுவிடம் இவ்விவகாரம் அனுப்பப்படும்.

## 24. நோய் குறித்து இரண்டாவது மருத்துவக்குழுவும் ஒருமித்த முறையில் இணக்கம் காணாவிட்டால் மருத்துவ முன் உத்தரவு நிறைவேற்றப்படுமா?

நோய் குறித்து இரண்டாவது மருத்துவக்குழுவும் ஒருமித்த முறையில் இணக்கம் காணாவிட்டால் மருத்துவ முன் உத்தரவு நிறைவேற்றப்படமாட்டாது. நோயாளிக்கு வழக்கமான சிகிச்சை அளிக்கப்படுவதோடு, அவருடைய உயிர் தொடர்ந்து காக்கப்படும்.

## 25. சுகாதார அமைச்சு நியமிக்கும் மூன்று பேர் அடங்கிய இரண்டாவது மருத்துவக் குழுவின் சேவைகளுக்காக நான் கட்டணம் செலுத்த வேண்டுமா?

அவர்களின் சேவைகளுக்காக நீங்கள் கட்டணம் செலுத்த வேண்டியதில்லை.

## மருத்துவ முன் உத்தரவில் நான் கையெழுத்து இட்ட பிறகு என்ன நடக்கும்?

### 26. மருத்துவ முன் உத்தரவில் நான் கையெழுத்து இட்டிருப்பது பற்றி என் மருத்துவர்களிடமோ வேறு யாரிடமோ தெரிவிக்க வேண்டுமா?

உங்கள் மருத்துவ முன் உத்தரவு ஏற்பாடு பற்றி எவரிடமும் சொல்ல வேண்டிய கட்டாயம் உங்களுக்கு இல்லை. ஆயினும், மருத்துவ முன் உத்தரவுக்கு முன்பு நீங்கள் உங்கள் குடும்பத்தினருடனும் நெருங்கியவர்களுடனும் கலந்தாலோசிப்பது நல்லது.

### 27. என் மருத்துவ முன் உத்தரவை யார் அறிவார்கள்?

உங்கள் மருத்துவ முன் உத்தரவு இரகசியமாக இருக்கும். அதைப் பற்றிக் கீழ் கண்டவர்களுக்கு மட்டும் தெரியப்படுத்தப்படும்.

i. நீங்கள் எழுத்து மூலம் அதிகாரம் அளித்துள்ள தனி நபர்களுக்கும்;
ii. இறப்பை ஏற்படுத்தும் நோயினால் நீங்கள் பாதிக்கப்பட்டுள்ளீர்கள் என்றும் உங்கள் விருப்பத்தை வெளியில் கூற உங்களால் இயலாது என்றும் நம்ப உங்கள் மருத்துவர்களுக்கு நியாயமான காரணம் இருந்தால் அவருக்கும்.

### 28. மருத்துவ முன் உத்தரவு பிறப்பித்துள்ள ஒரு நோயாளி மரணக் கட்டத்தில் இருந்தால் அவர் மருத்துவ சிகிச்சையின்றி அல்லது பட்டினியால் அவதிப்படுமாறு விடப்படுவாரா?

மரணக் கட்டத்தில் இருக்கும் நோயாளிக்கு மருத்துவர்கள் தொடர்ந்து சிகிச்சை அளித்து அவருடைய வலியையும் அவதியையும் போக்க உதவுவார்கள்.
மருத்துவ முன் உத்தரவு இருந்தாலும் கூட, தொடர்ந்து மருந்து கொடுக்கப்படும்; நோயின் கடுமை தணிக்கப்படும்.

### 29. நோய்த்தணிப்பு பராமரிப்பு என்பது என்ன?

உயிர்கொல்லிநோயால் அவதிப்படும் நோயாளிகளுக்கும் அவர்கள் குடும்பத்தினருக்கும் ஏற்படும் மற்ற சிரமங்கள், உடல், மனம் மற்றும் ஆன்மிகம் சம்பந்தப்பட்ட பிரச்சனைகள் வராமல் தடுத்து, அவற்றை ஆராய்ந்து, சிகிச்சை அளித்து, அவர்களின் வாழ்க்கைத் தரத்தை மேம்படச் செய்வதே, நோய்த்தணிப்பு பராமரிப்பு எனப்படும் பராமரிப்பு முறை.

மருத்துவ முன் உத்தரவு சட்டத்தின்படி நோய்த்தணிப்பு பராமரிப்பு முறையில் உள்ள அம்சங்கள்:

- வலி, வேதனை, அசௌகரியம் ஆகியவற்றின் நிவாரணத்திற்கு ஏற்ற மருத்துவ சிகிச்சை வழிமுறைகளை வழங்குதல்; மற்றும்
- ஏற்ற அளவு தண்ணீர் மற்றும் உணவை வழங்குதல்.

### 30. மருத்துவ முன் உத்தரவு நிறைவேற்றப்படுவதற்கு முன் எனக்குத் தேவைப்படும் எல்லா சிகிச்சையும் வழங்கப்பட்டிருப்பதை உறுதிப்படுத்த என்ன பாதுகாப்பு முறைகள் இருக்கின்றன?

பிணியில் மரணக்கட்டத்தில் நோயாளி இருக்கிறார் என்று உறுதிப்படுத்தப்படாத நிலையில் அவர் குணமடையும் சாத்தியம் இருப்பதால், அவருக்கு தேவையான எல்லா சிகிச்சையும் வழங்கப்படுவதை உறுதிப்படுத்துவது மருத்துவரின் தொழில்முறைப் பொறுப்பு. மரணக்கட்டத்தில் நோயாளி இருக்கிறார் என்று கருதப்பட்டாலும் அவருடைய வலியையும் அவதியையும் குறைக்க சிகிச்சை அளிக்கும் பொறுப்பு மருத்துவருக்கு உண்டு.

இது நடப்பில் உள்ள முறை. நோயாளி மருத்துவ முன் உத்தரவு பிறப்பித்தாலும் பிறப்பிக்காவிட்டாலும் இந்த நடைமுறை பின்பற்றப்படும்.

## மருத்துவ முன் உத்தரவை ரத்து செய்தல்

### 31. நான் ஏற்கனவே பதிந்த மருத்துவ முன் உத்தரவு தொடர்பில் எனக்கு மனமாற்றம் ஏற்பட்டால் அதை நான் ரத்து செய்ய முடியுமா?

மருத்துவ முன் உத்தரவை எப்போது வேண்டுமானாலும் ரத்து செய்யலாம். ஆனால் அதற்கு ஒரு சாட்சியாவது உடனிருக்க வேண்டும். மருத்துவ முன் உத்தரவை ரத்து செய்பவர், படிவம் 3 மூலம் இதைச் செய்ய வேண்டும். *(மருத்துவ முன் உத்தரவைப் பிறப்பித்து உள்ளவர்கள் படிவம் 2 உடன், படிவம் 3-யும் பெறுவார்கள். மருத்துவ முன் உத்தரவுப் பதிவகத்தில் முன் உத்தரவு பதியப்பட்டிருப்பதை படிவம் 2 உறுதிப்படுத்துகிறது.)*

மாறாக, அந்த நபரோ அல்லது அவரது சாட்சியோ மருத்துவ முன் உத்தரவு பதிவாளருக்கு கடிதம் எழுதலாம்.

**கீழ்க்காணும் தகவல் கடிதத்தில் இருக்க வேண்டும்:**

(a) மருத்துவ முன் உத்தரவை ரத்து செய்பவர்,சாட்சி ஆகியோருடைய பெயர், அடையாள அட்டை எண், முகவரி,வீட்டு, அலுவலகத் தொலைபேசி எண்கள்
(b) ரத்து செய்யப்பட்ட நேரம்,நாள், இடம்.
(c) அந்த நபர் மருத்துவ முன் உத்தரவை ரத்து செய்ய பயன்படுத்திய தொடர்பு முறை. (எ.கா. வாய்மொழி, சைகை மொழி ஆகியவை) அப்படி, அந்தக் கடிதத்தை நபரின் சாட்சி எழுதியிருக்கும் வேளையில்.

மருத்துவ முன் உத்தரவின் ரத்தை உடனடியாக மருத்துவ முன் உத்தரவு பதிவகத்திற்கு அனுப்ப வேண்டும்.

### 32. நான் எழுத முடியாத நிலையில் இருந்தால் என்ன செய்வது?

இந்தச் சூழ்நிலையில் பேச்சு மூலம் அல்லது நீங்கள் தகவல் தெரிவிக்கக் கூடிய வேறு எந்த வழியிலும் மருத்துவ முன் உத்தரவை ரத்து செய்யலாம். மேலே குறிப்பிட்டவாறு ரத்துப் படிவம் 3 அல்லது கடிதம் மூலம் ரத்து அறிவிப்பைச் சமர்ப்பிப்பது சாட்சியின் பொறுப்பாகும். **உத்தரவை நீக்குகிறவர் ஏன் தாமாகவே அதைச் செய்யவில்லை என்பதற்கான காரணத்தைச் சாட்சி கூற வேண்டும்.**

*பிரிவு 7(4) எழுத்து மூலமோ பேச்சு மூலமோ வேறு எந்த வகையிலோ ஒரு நோயாளி உத்தரவை ரத்து செய்வதற்குச் சாட்சியாக இருப்பவர் அது குறித்துப் பதிவாளரிடம் தெரிவிப்பது அவருடைய பொறுப்பு.*

## மருத்துவ முன் உத்தரவை நான் பிறப்பிக்கவில்லை என்றால்?

மருத்துவ முன் உத்தரவை நீங்கள் பிறப்பிக்க விரும்பவில்லை என்றால் அவ்வாறு பிறப்பிப்பது கட்டாயம் அல்ல. மருத்துவ முன் உத்தரவைப் பிறப்பிக்காத, இறப்பை ஏற்படுத்தும் நோயினால் பாதிக்கப்பட்ட ஒருவர், சுய நினைவை இழந்து, தன் விருப்பத்தை வெளியில் கூற இயலாத நிலையில் இருந்தால் நடப்பிலுள்ள நடைமுறை தொடரும். மருத்துவர் நோயாளியின் குடும்ப உறுப்பினர்களுடன் கலந்தாலோசித்த பின், நோயாளிக்கான சிகிச்சை பற்றி அவர்கள் முடிவு செய்வார்கள்.

மருத்துவமனையில் சேர்வதற்கு மருத்துவ முன் உத்தரவு ஒரு முன் தேவை **அல்ல** என்பதை **நினைவில்** கொள்ளுங்கள். சட்டப்படி, அது காப்புறுதி எடுப்பதைப் பாதிக்காது.

## மருத்துவ முன் உத்தரவு படிவத்திற்கான குறிப்புகள்

1) மருத்துவ முன் உத்தரவை சமர்ப்பிப்பதற்கான படிவம் (படிவம் 1) இந்தக் கையேட்டின் இறுதியில் உள்ளது. முதல் சாட்சியாக விளங்கவிருக்கும் உங்கள் மருத்துவரிடம் இருந்தும் படிவத்தை நீங்கள் பெற்றுக் கொள்ளலாம்:சுகாதார அமைச்சின் இணையதளத்தில் இருந்தும் படிவத்தை மின்னியல் இறக்கம் செய்துகொள்ளலாம். *http://moh.gov.sg/amdform*

2) உங்கள் மருத்துவர், மருத்துவ முன் உத்தரவின் பாதுகாப்பளிக்கும் முறைகள், அதற்கு பின்பற்றவேண்டிய வழிமுறைகள் போன்றவற்றோடு, எப்போது உங்கள் மருத்துவ முன் உத்தரவு பயன்படுத்தப்படும் போன்ற விவரங்களையும் உங்களுக்கு விளக்குவார்.

3) நீங்கள் 21 வயதுக்கு மேற்பட்டவரா, தன்னிச்சையாக கையெழுத்திடுகிறீர்களா, கையெழுத்திடுவதால் ஏற்படும் விளைவுகளை நன்கு புரிந்து கொண்டிருக்கிறீர்களா, தெளிவான சிந்தனையில் இருக்கிறீர்களா போன்றவற்றையும் உங்கள் மருத்துவர் மறு உறுதி செய்து கொள்வார்.

   நீங்கள் கையெழுத்திடுவதற்கான காரணங்கள், விளக்கங்கள், விளைவுகள் ஆகியவற்றை நன்கு புரிந்து கொண்டிருப்பதும், உங்கள் பெயர், உங்கள் குடும்பத்தாரின் பெயர்கள், நீங்கள் அந்த சமயத்தில் இருக்கும் இடம், நேரம் போன்றவற்றை குறிப்பிடுவதும் உங்களின் தெளிவான சிந்தனைக்கு அடையாளமாக விளங்கும்.

4) நீங்களும், உங்கள் இரு சாட்சிகளும் மருத்துவ முன் உத்தரவு படிவத்தில் கையெழுத்திட்ட பிறகு, இந்தக் கையேட்டின் இறுதியில் இணைக்கப்பட்டுள்ள முத்திரை பதித்த கடித்த உறையில், மருத்துவ முன் உத்தரவு பதிவகத்திற்கு அனுப்பவும்.

5) உங்கள் படிவத்தை மருத்துவ முன் உத்தரவு பதிவகம் பெற்றுக்கொண்டதும் உங்களுக்கு ஒரு பதில் கடிதம் **(படிவம் 2)** அனுப்பப்படும். மருத்துவ முன் உத்தரவை பதிவகத்தில் நீங்கள் பதிந்துகொண்டதை அது உறுதி செய்யும்.

இந்த மருத்துவ முன் உத்தரவு பற்றிய மேல் விவரங்களுக்கு சுகாதார அமைச்சை 63259136 என்ற அவசர தொலைபேசி எண் மூலம் தொடர்பு கொள்ளலாம்.அல்லது, சுகாதார அமைச்சின் இணையபக்கத்தற்கும் செல்லலாம்:

*http://www.moh.gov.sg/amd*

Serial No: ____________
(for official use)

**FORM 1**

# MAKING OF ADVANCE MEDICAL DIRECTIVE

THE ADVANCE MEDICAL DIRECTIVE ACT 1996 [ACT 16 OF 1996, SECTION 3]

THE ADVANCE MEDICAL DIRECTIVE REGULATIONS 1997

(This form may take you 5 minutes to fill in)

**PERSON MAKING THE ADVANCE MEDICAL DIRECTIVE**

Name: ______________________

NRIC No.: __ - _______ - __ Sex: ☐ Male ☐ Female (please tick)

Date of Birth: __ - __ - __ (must be at least 21 years of age)
Day Month Year

Address: ______________________

______________________ Singapore ______

Home Telephone: ________ Office Telephone: ________

## THE DIRECTIVE

1. I hereby make this advance medical directive that if I should suffer from a terminal illness and if I should become unconscious or incapable of exercising rational judgment so that I am unable to communicate my wishes to my doctor, no extraordinary life-sustaining treatment should be applied or given to me.

2. I understand that "terminal illness" in the Advance Medical Directive Act 1996 means an incurable condition caused by injury or disease from which there is no reasonable prospect of a temporary or permanent recovery where -
   (a) death would within reasonable medical judgment be imminent regardless of the application of extraordinary life-sustaining treatment; and
   (b) the application of extraordinary life-sustaining treatment would only serve to postpone the moment of death.

3. I understand that "extraordinary life-sustaining treatment" in the Advance Medical Directive Act 1996 means any medical procedure or measure which, when administered to a terminally ill patient, will only prolong the process of dying when death is imminent, but excludes palliative care.

4. This directive shall not affect any right, power or duty which a medical practitioner or any other person has in giving me palliative care, including the provision of reasonable medical procedures to relieve pain, suffering or discomfort, and the reasonable provision of food and water.

5. I make this directive in the presence of the two witnesses named on page 2.

Signature / Thumb Print                                        Date

**INSTRUCTIONS ON THE REGISTRATION OF ADVANCE MEDICAL DIRECTIVE**

1. The person making the advance medical directive should complete this form and send it in a sealed envelope by mail or by hand to the Registrar of Advance Medical Directives at the address given below. Faxed copies will not be accepted.

2. The advance medical directive is only valid when it is registered with the Registrar of Advance Medical Directives. The Registrar will send the maker of the directive an acknowledgement when the directive has been registered.

**The Registry of Advance Medical Directives**
Ministry of Health, College of Medicine Building, 16 College Road, Singapore 169854
Tel: 63259136 Fax: 63259212

(Please direct all enquiries to this address)



Glue Here

Glue Here

Glue Here

(Both witnesses please read the *NOTES FOR WITNESS* below before signing)

**NOTES FOR WITNESS**

A witness shall be a person who to the best of his knowledge -
(a) is not a beneficiary under the patient's will or any policy of insurance;
(b) has no interest under any instrument under which the patient is the donor, settlor or grantor;
(c) would not be entitled to an interest in the estate of the patient on the patient's death intestate;
(d) would not be entitled to an interest in the moneys of the patient held in the Central Provident Fund or other provident fund on the death of that patient; and
(e) has not registered an objection under section 10(1) of the Advance Medical Directive Act 1996.

**FIRST WITNESS** (This witness must be a registered medical practitioner)

Name:

NRIC No.: - -

Office Address:

Singapore

Office Telephone: Handphone / Pager: 9 -

1. I have taken reasonable steps in the circumstances to ensure that the maker of this directive -
   (a) is of sound mind;
   (b) has attained the age of 21 years;
   (c) has made the directive voluntarily and without inducement or compulsion; and
   (d) has been informed of the nature and consequences of making the directive.
2. I declare that this directive is made and signed in my presence together with the witness named below.

Signature of the Medical Practitioner | Name/Clinic Stamp of the Medical Practitioner | Date

Note: As a *guide* for the purposes of determining whether the maker of the directive is of sound mind, the medical practitioner should ascertain whether the maker -
(a) understands the nature and implications of the directive;
(b) is oriented to time and space; and
(c) is able to name himself and his immediate family members.

**SECOND WITNESS** (This witness must be of at least 21 years of age)

Name:

NRIC No.: - -

Home Address:

Singapore

Home Telephone: Office Telephone:

I declare that this directive is made and signed in my presence together with the witness named above.

Signature | Date



**CONFIDENTIAL**
**TO BE OPENED BY THE REGISTRY ONLY**

Postage will be paid by addressee.
For posting in Singapore only

**BUSINESS REPLY SERVICE**
**PERMIT NO. 08081**

**The Registry of Advance Medical Directives**
Ministry of Health, College of Medicine Building,
16 College Road, Singapore 169854

For further queries, please contact:
**Registry of Advance Medical Directives**
Ministry of Health
College of Medicine Building
16 College Road,
Singapore 169854
Tel: 6325 9136
Fax: 6325 9212
*Internet Email: MOH_INFO@MOH.GOV.SG*

MINISTRY OF HEALTH
SINGAPORE
