# Supplementary material for: Increasing the completion rate of the advance directives in primary care setting – a randomized controlled trial
Source: BMC Fam Pract. 2021 Jun 18;22:115. doi: 10.1186/s12875-021-01473-1 (PMC8214280; doi:10.1186/s12875-021-01473-1)
Supplement: Supplementary file 4 — Additional file 4. [file 12875_2021_1473_MOESM4_ESM.docx]

***Table S1 Reasons for ADs amongst participants who completed / planned to complete an ADs* *at 6 weeks follow-up (n=57)***

|  | **Previous experience with someone close with terminal illness** | **Had prior discussion with family** | **Belief that passing away from a terminal illness if better than an artificially prolonged life** | **Wish to lessen financial burden** | **Wish to lessen emotional burden** | **Wish to avoid prolonged suffering** | **Acceptance of death in terminal illness** |
| --- | --- | --- | --- | --- | --- | --- | --- |
| **Yes** | 33(57.9%) | 33(57.9%) | 45(78.9%) | 28(49.1%) | 42(73.7%) | 47(82.5%) | 44(77.2%) |
| **No** | 10(17.5%) | 11(19.3%) | 0 (0.0%) | 2(3.5%) | 0(0.0%) | 0(0.0%) | 0(0.0%) |
| **Neutral** | 14(24.6%) | 13(22.8%) | 12(21.1%) | 27(47.4%) | 15(26.3%) | 10(17.5%) | 13(22.8%) |
