# Supplementary material for: Increasing the completion rate of the advance directives in primary care setting – a randomized controlled trial
Source: BMC Fam Pract. 2021 Jun 18;22:115. doi: 10.1186/s12875-021-01473-1 (PMC8214280; doi:10.1186/s12875-021-01473-1)
Supplement: Supplementary file 5 — Additional file 5. [file 12875_2021_1473_MOESM5_ESM.docx]

|  | **Bad luck to talk about death** | **Too young to be concerned about death** | **Unlikely to be in a situation that required an AMD** | **Inconvenience** | **Goes against religious beliefs** | **Goes against personal ethics** | **Family members** | **Mistrust in doctors** | **Terminology unclear** |
| --- | --- | --- | --- | --- | --- | --- | --- | --- | --- |
| **Yes** | 17(21.8%) | 23(29.5%) | 37(47.4%) | 35(44.9%) | 14(18.0%) | 6(7.6%) | 20(25.6%) | 3(3.9%) | 10(12.8%) |
| **No** | 27(34.6%) | 25(32.1%) | 11(14.1%) | 14(17.9%) | 32(41.0%) | 36(46.2%) | 28(35.9%) | 43(55.1%) | 24(30.8%) |
| **Neutral** | 34(43.6%) | 30(38.4%) | 30(38.5%) | 29(37.2%) | 32(41.0%) | 36(46.2%) | 30(38.5%) | 32(41.0%) | 44(56.4%) |

***Table S2 Reasons against ADs amongst participants who did not complete / plan to complete an AD at 6 weeks follow-up (n=78)***
